# Modification of the Transcription Factor FOXL2 at Serines 101 and 107 Disables DNA Binding, Leads to Nucleolar Relocalization, and Rewires Granulosa‐Cell Programs

**DOI:** 10.1096/fj.202602528R

**Published:** 2026-08-01

**Authors:** Ludovic Mousseron, Despoina Chousianiti, Francis Poulat, Bérangère Legois, Reiner A. Veitia, Anne‐Laure Todeschini

**Affiliations:** ^1^ Université Paris Cité, CNRS, Institut Jacques Monod Paris France; ^2^ Institute of Human Genetics, CNRS UMR9002 University of Montpellier Montpellier France; ^3^ Université Paris Saclay Paris France; ^4^ Institut de Biologie François Jacob, CEA Paris France

## Abstract

FOXL2 is a forkhead transcription factor (TF) essential for granulosa‐cell identity and function, yet how post‐translational modifications tune its activity remains incompletely understood. Here, we show that protein kinase C (PKC) phosphorylates FOXL2 in vitro. Two of the four phosphorylation sites, notably Ser101 and Ser107, map to the forkhead DNA‐recognition helix. Phosphomimetic substitutions (S → D) at these positions (S101D/S107D) abolish binding to a consensus DNA sequence recognized by FOXL2 and luciferase reporter activation, whereas alanine substitutions are rather neutral. In HeLa cells, the S101D mutant and, to a lesser extent, S107A/S107D, relocalize at least partially to nucleoli and exhibit increased mobility consistent with reduced DNA engagement. This pattern was recapitulated in stably transduced KGN granulosa cells. RNA‐seq of such KGN cells revealed that S101D and a C‐terminal truncation (ΔC) induce a massive loss‐of‐function (LOF) relative to wild‐type (WT) FOXL2. The LOF affects sets of genes involved in pathways central to granulosa physiology including ECM organization, cell migration/adhesion, and MAPK cascades, whereas S101A is largely WT‐like. An analysis of the FOXL2 interactome in the transduced cells by mass spectrometry (MS) showed that S101D loses numerous interactions with TFs and chromatin remodelers, and Pol I/III regulators such as UBTF and TFIIIC components, while it gains other partners. By contrast, ΔC retains many of the protein–protein contacts of WT and preferentially loses ribosomal/TFIII interactions. Together, these data allow us to hypothesize that PKC‐dependent phosphorylation within the FOXL2 DNA‐recognition helix would underlie a rapid, reversible switch, weakening DNA binding, redirecting subnuclear partitioning, and rewiring protein–protein interactions, thereby reshaping FOXL2‐dependent gene regulation in granulosa cells.

## Graphical abstract

PKC‐mediated phosphorylation of FOXL2 weakens DNA binding, alters subnuclear localization and protein interactions, and reshapes FOXL2‐dependent transcriptional programs in granulosa cells, supporting a reversible mechanism that modulates FOXL2 activity through phosphorylation of its DNA‐binding domain.

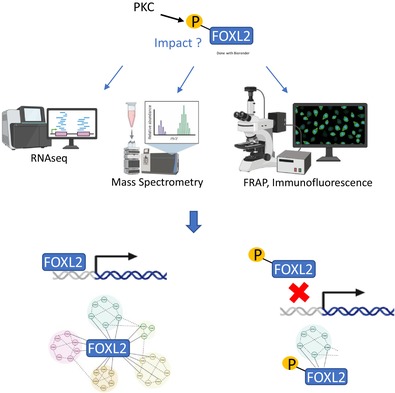

## 1 Introduction

FOXL2 is a transcription factor (TF), which is highly conserved across vertebrates. It belongs to the FOX family of TFs, with which it shares a highly conserved forkhead DNA‐binding domain (FHD). The FHD is characterized by a 3D structure arranged as a winged helix domain [1]. FOXL2 is mainly expressed in the eyelids, the pituitary gland, and in the granulosa cells (GCs) of the ovary [2, 3]. Its pathogenic variants are responsible for the Blepharophimosis Ptosis Epicanthus Inversus syndrome (BPES), which results in palpebral malformations often associated with female infertility [4]. Besides, a somatic missense variant (p. C134W) has been identified in most adult‐type granulosa‐cell tumors (AGCTs) [5].

FOXL2 is involved in numerous cellular processes, such as apoptosis, regulation of cell proliferation, cell cycle control, steroidogenesis, and reactive oxygen species detoxification [6, 7]. The constitutive knock‐out (KO) of *Foxl2* leads to massive follicular atresia [8, 9], whereas its conditional KO results in a “masculinization” of the ovary. Indeed, the absence of FOXL2 leads to a transdifferentiation of murine adult granulosa cells into Sertoli‐like cells and to the de‐repression of “male‐pathway” genes, such as *SRY‐box transcription factor 9 (Sox9)* [10]. This shows that FOXL2 is essential for ovarian development and maintenance as a repressor of the genes responsible for testis differentiation throughout life.

Numerous post‐translational modifications (PTMs) of FOXL2 have been reported, such as acetylation, SUMOylation, and phosphorylation, among others [11]. These modifications have been proposed to modulate FOXL2 activity by affecting its localization, stability, DNA binding, or protein–protein interactions (PPIs). For instance, SUMOylation of FOXL2 increases its stability and leads to its recruitment to Promyelocytic leukemia protein (PML) bodies [11]. Several FOX TFs, such as FOXO1, FOXO3, and FOXO4, have been shown to be regulated by phosphorylation [12]. FOXO proteins are phosphorylated by a large number of protein kinases (PK), including AKT/PKB, SGK, and AMPK [13, 14, 15, 16, 17, 18, 19]. Phosphorylation of FOXO by AKT was one of the first to be studied, and it was shown that this PK phosphorylates the FOXOs on 3 residues, leading to their sequestration in the cytoplasm. In contrast, phosphorylation by JNK or MST1 increases FOXO transcriptional activity [17, 20, 21, 22]. Unlike FOXO, little is known about FOXL2 phosphorylation, the relevant PKs, and the effects of this PTM on its activity. In a previous study, LATS1 was found to phosphorylate FOXL2 on a serine, the position of which is yet to be identified [23]. Another study showed that GSK3B interacts with FOXL2 and phosphorylates it at Ser33. Interestingly, this serine residue is found hyperphosphorylated in AGCTs [24] and leads to the polyubiquitination of FOXL2 at Lys25, resulting in its degradation by the proteasome.

Here, we set to study FOXL2 phosphorylation and more specifically by protein kinase C (PKC), which is known to play a key role in follicle development and fate. Indeed, the effect of Follicle‐stimulating hormone (FSH) on follicular growth has long been attributed to PKA, but recent studies have shown that PKA and PKC cooperate in response to the binding of FSH to its receptor [25, 26, 27]. Furthermore, PKC signaling plays an important role in mediating the effect of Luteinizing hormone (LH) on granulosa cell differentiation and follicular maturation, eventually leading to ovulation [28].

In this study, we demonstrate that FOXL2 is phosphorylated by PKC in vitro on several residues and that these modifications alter FOXL2 localization, activity, and PPIs. Specifically, phosphorylation of the FHD residues Ser101 and Ser107 by PKC is expected to lead to major changes in FOXL2. Consistently, the phosphomimetic substitutions S101D and S107D abolish DNA binding and drive nucleolar enrichment. RNA‐seq shows that S101D and a C‐terminal truncation (ΔC) produce a major LOF, indicating that DNA engagement and the C‐terminus are required for full transcriptional output. By contrast, S101A perturbs the expression of a few transcripts. Interactome profiling mirrored these trends. Together, our data support a model in which PKC‐dependent phosphorylation within the FOXL2 DNA‐recognition helix would act as a rapid, reversible switch that weakens DNA binding, redirects subnuclear partitioning toward nucleoli, and rewires PPIs, thereby changing FOXL2‐dependent transcriptional programs.

## 2 Material and Methods

### 2.1 Cell Culture

HeLa (RRID:CVCL_0030) and KGN WT/WT‐FOXL2 cells [29] were maintained in DMEM‐F12 medium supplemented with GlutaMAX (Gibco, 31331028) and with 10% fetal bovine serum (FBS, Gibco‐Invitrogen, A5256701) plus 1% penicillin/streptomycin (Gibco, 15140122). These cell lines were derived from female patients. Cells were cultured at 37°C in a humid atmosphere with 5% CO_2_. For the different assays, cells were seeded as follows: in 24‐well plates containing sterile coverslips for phenotype identification by microscopy, in 96‐well plates for luciferase assays, in 6‐well plates for RNA extraction, and in 150 cm^2^ flasks for protein extraction for MS.

### 2.2 Cloning in Expression Vectors

The FOXL2‐WT‐3xFLAG and FOXL2‐WT‐GFP expression vectors were those previously described [11, 30]. Plasmids carrying the A/D substitution mutants at serines S101 and S107 were generated by fusion PCR as previously described [31]. They were sequence‐verified prior to use. For subcloning into GFP and 3XFLAG expression vectors, the FOXL2 variants coding sequence was amplified by PCR to introduce EcoRV and KpnI restriction sites. Both the amplified inserts and the pcDNA3.1‐GFP/pcDNA3.1‐3XFLAG vectors were then digested with EcoRV and KpnI restriction enzymes. Ligation was performed with a vector/insert ratio of 1/3 and with T4 DNA ligase (Biolabs, M0202S).

### 2.3 Lentiviral Preparation and Cells Transduction

Doxycycline‐inducible FOXL2 cells were generated using the Lenti‐X TetOne Inducible Expression System (Clonetech, 631844), which allows controlled expression of WT FOXL2 and FOXL2 variants protein (Doxycycline from Sigma‐Aldrich, D9891). The coding sequences of WT FOXL2 and its variants tagged with 3XFLAG were subcloned using EcoRI and BamHI into the lentiviral vector pLVX‐TetOne‐Puro (RRID:Addgene_171123).

Lentiviruses were produced in 293T cells (ATCC CRL‐3216, RRID:CVCL_0063) plated in 175 cm^2^ flasks with a density of 9000 cells per cm^2^ and cultured in DMEM (4.5 g/L glucose + GlutaMAXTM Supplement+ pyruvate) + 1% penicillin–streptomycin + 10% FCS for 72 h. Cells were co‐transfected with packaging plasmids (70 μg of pCMV‐VSV‐G: Addgene #8454 (RRID:Addgene_8454) and 100 μg of pCMVΔR8.91 [32]) and 100 μg of expression vector using the calcium phosphate method [33]. Transfected cells were incubated for 17 h at 37°C with 5% CO_2_, after changing the culture medium, cells cultured for additional 48 h. Then, the culture medium containing viral particles was filtered using 0.45 μm polyethersulfone filter (Nalgene Rapid‐Flow membrane PES 0.45 μm 50 mm, 115 mL, 165‐0045). The viral suspension was centrifuged at 83 000 *g* for 90 min at 4°C. Finally, the pellet was suspended in 200 μL PBS and frozen at −80°C.

To generate the KGN cells expressing the different FOXL2 variants, KGN WT/WT for FOXL2 cells were seeded in 6‐well plates at 50% confluence 24 h prior to transduction. A range of virus quantities was tested to determine that with the fewest cells surviving after selection to ensure a minimal viral integration event. Twenty‐four hours after transduction, cells were washed three times with dPBS (Gibco, Dulbecco PBS, no calcium, no magnesium, 14190094) to remove residual viral particles, then the cells were trypsinized (Gibco, Trypsin–EDTA (0.05%), phenol red, 25300054) and transferred to 75 cm^2^ flasks. Stable polyclonal cell populations were obtained by selection with 1 μg/mL puromycin (Gibco, A11138‐03). Once confluence was reached, cells were transferred to 150 cm^3^ flasks for maintenance.

### 2.4 In Vitro Phosphorylation Assays and MS

In vitro phosphorylation assays were performed using the Kinase Enzyme System Kits (Promega Corporation, USA): AKT1 (A16‐10G), GSK3β (G09‐10G), PKA (V4246), PKCα (P61‐18G) according to the manufacturer's instructions. For FOXL2 we used 1 μg of purified (bacterially expressed) protein, conserved in 20 mM Tris–HCl pH 8, 272 mM NaCl, 1 mM DTT buffer. The volume for the phosphorylation reaction was adjusted with water to 12.5 μL. After 1 h of incubation at room temperature with gentle agitation, 12.5 μL of ADP‐Glo solution (Promega, V6930) and the mixture was incubated for 40 min at room temperature with gentle agitation (removal of ATP in excess). Next, we added 25 μL of kinase activity detection solution and incubated the samples for 30 min at room temperature in the dark with gentle stirring. Light emission was measured using a LB 960 luminometer (Berthold Technologies, Bad Wildbad, Germany).

For MS we performed a similar reaction with 10 μg of purified WT FOXL2 in 25 μL.

For intact mass analysis, samples were injected in microliter pickup mode onto a custom made BioResolve RP (450 Å, 2.7 μm, 0.3 mm × 150 mm) Polyphenyl Column (Waters, Milford, MA, USA) using an UltiMate 3000 RSLCnano chromatographic system fitted with a capillary flow meter from ThermoFisher Scientific (Waltham, MA, USA). Mobile phases A and B were respectively H2O/acetonitrile (ACN) 98/2 and H2O/ACN 20/80 acidified with 0.1% (v/v) formic acid (FA). The sample was eluted at a flow rate of 6 μL/min using the following slope change points: 0–5 min hold at 6.3% B, 5 min slope until 31.3% B, 40 min slope until 56.2% B, 5 min wash at 100% B and finally 10 min hold at 6.3% B. The eluent was sprayed using the conventional ESI ion source of a Q‐Exactive Plus mass spectrometer from ThermoFisher Scientific operating in the positive ion mode. MS spectra were acquired at a resolution of 70K, with a mass range of m/z 500–2500 and an AGC target value of 1e6 for a maximum ion accumulation time of 500 ms. Mass spectra were deconvoluted using the Intact software from Protein Metrics (RRID:SCR_006944, Boston, MA, USA).

For LC–MS/MS analysis, proteins were precipitated by adding six volumes of cold acetone (−20°C). After vortexing, samples were incubated overnight at −20°C and centrifuged for 10 min at 11 000 rpm, 4°C. The supernatant was discarded, and protein pellets were resuspended in 8 M urea, 25 mM ammonium bicarbonate. Proteins were reduced with 10 mM DTT (Sigma‐Aldrich) and alkylated with 20 mM iodoacetamide (IAA) (Sigma‐Aldrich). After a 16‐fold dilution in 25 mM ammonium bicarbonate, samples were digested overnight at 37°C using either trypsin (Promega) or chymotrypsin (Promega) at an enzyme‐to‐substrate ratio of 1:10. The resulting peptides were desalted on Evotips Pure (Evosep One, Odense, Denmark) according to the manufacturer. Samples were analyzed on a timsTOF Pro 2 mass spectrometer (Bruker Daltonics, Bremen, Germany) coupled to an Evosep one system (Evosep, Odense, Denmark) operating with the 30SPD method developed by the manufacturer. Briefly, the method is based on a 44‐min gradient and a total cycle time of 48 min with a C18 analytical column (0.15 × 150 mm, 1.9 μm beads, ref. EV‐1106) equilibrated at 40°C and operated at a flow rate of 500 nL/min. H2O/0.1% FA was used as solvent A and ACN/0.1% FA (ThermoFisher Scientific) as solvent B. The timsTOF Pro 2 was operated in PASEF mode 1 over a 1.3 s cycle time. Mass spectra for MS and MS/MS scans were recorded between 100 and 1700 *m/z*.

MS raw files were processed using PEAKS Studio (Bioinformatics Solutions Inc.). Data were searched against the SwissProt *Homo Sapiens* database (downloaded January 2023). Parent mass tolerance was set to 20 ppm, with fragment mass tolerance to 0.05 Da. Specific cleavages were selected and a maximum of 2 missed cleavages were allowed. The following PTM were considered for identification: Oxidation (M), Deamidation (NQ) and Phosphorylation (STY) as variables and carbamidomethylation (C) as fixed. Identifications were filtered based on a 1% FDR (False Discovery Rate) threshold at both peptide and protein group levels.

### 2.5 Luciferase Assay

Luciferase assays were performed in HeLa cells. Cells were seeded at 25% confluence in 96‐well plates and transfected with 40 ng/well of reporter plasmid (GRAS‐luc, p16‐luc, dAT29C‐luc), 10 ng/well of transfection control (Renilla luciferase), and 25 ng/well of each expression plasmid using 0.45 μL/well Lipofectamine 2000 reagent (Invitrogen, 11668027). After 48 h, cells were lyzed and analyzed with the Dual‐Glo Luciferase Assay System (Promega, E2920) according to the manufacturer's instructions. Relative luciferase activity was determined as the ratio of firefly to Renilla luciferase. Luminescence was measured using a LB 960 luminometer (Berthold Technologies, Bad Wildbad, Germany). Reported values represent the mean of six biologically independent replicates. Statistical significance was measured using a Tukey test, and error bars indicate standard deviation. We used GraphPad Prism (RRID:SCR_002798) to perform the analysis.

### 2.6 Preparation of Probes and Gel‐Shift Assay

The oligos containing the forkhead consensus binding sequence (5′‐ggTCGCAACACTTGTTTACATTCTCCAACC‐3′, 5′‐ggGGTTGGAGAATGTAAACAAGTGTTGCGA‐3′) were annealed by heating to 80°C for 3 min in 20 mM Hepes‐KOH [pH 7.9], 50 mM NaCl, and 6 mM MgCl2; slow‐cooled to 50°C; held at that temperature for ten minutes and then cooled to room temperature. The duplexes were de‐salted through a Sephadex G‐50 spin column (cytiva ref. 27533001). The de‐salted duplexes (100 ng) were labeled with reverse transcriptase Superscript 2 or 3 and α [32P]‐dCTP (10 μCi) for 1 h. The duplexes/probes were then purified to remove free α [32P]‐dCTP with Sephadex G‐50 spin column. Unlabelled FOXL2 proteins were produced using the TNT Quick Coupled transcription/translation system (Promega) according to the manufacturer's instructions. As a control of translation efficiency, TnT reactions were performed in presence of 35S‐methionine. DNA binding reactions contained 10 mM Tris–HCl (pH 7.9), 100 mM KCl, 10% glycerol, 5 mM MgCl2, 1 mM spermidine, 0.075% Triton‐X‐100, 1 mM DTT, 1 μg BSA, 200 μM ZnSO4, 0.1 μg dI‐dC, 0.5 ng radio‐labeled duplex probe, and 2 μL in vitro translation mix, in a total volume of 20 μL.

Binding reactions were performed on ice for ten minutes and complexes were resolved on a 5% native acrylamide (37.5:1) gel in 0.5× TBE at 120 V. Gels were dried and analyzed using a Typhoon PhosphoImager from Amersham.

### 2.7 FOXL2 Subcellular Localisation in HeLa Cells

HeLa cells were seeded on sterile glass coverslips in 24‐well plates transfected using the calcium phosphate method with plasmids encoding WT FOXL2 or the variants (1250 ng/well). Twenty‐four hours post‐transfection, cells were washed for 1 min with dPBS1X + EGTA 5 mM followed by a wash with dPBS1X and further incubated for an additional 24 h before fixation. Cells were washed with dPBS1X, then fixed with 4% PFA/H_2_O for 15 min at room temperature, then washed twice with dPBS1X. Nuclear and nucleolar staining was performed for 45 min at room temperature using Hoechst 10 μg/mL (Invitrogen, 33342) and 5 μmol/L (DOJINDO, N512‐10) Nucleolus Bright Red in dPBS1X, respectively. Cells were washed twice with PBS1X, then the coverslips were mounted on glass slides using Fluoromount G (Invitrogen, 00‐4958‐02). Fluorescence images were obtained using a CSU‐X1 spinning disk microscope equipped with 63×/1.4 Plan‐Apochromat Oil DIC (Zeiss, 420782‐9900) 0.103 μm/pixel, Image acquisition was performed with ZEN blue 2.6 software.

### 2.8 Immunofluorescence in KGN Cells

KGN cells were seeded on sterile glass coverslips in 24‐well plates at 25% confluence, 24 h prior to transduction. Cells were then transduced with pLVX‐FOXL2‐WT‐3xFLAG lentiviruses or FOXL2 variants, without antibiotic selection. 24 h after transduction, cells were treated with 500 ng/mL doxycycline for an additional 24 h before slide mounting. Cells were washed with dPBS1X then fixed with 4% PFA/H_2_O for 10 min at room temperature. After fixation, cells were washed three times with dPBS1X and permeabilized with 0.2% Triton X‐100/PBS1X for 10 min. Cells were then washed three times with dPBS1X. Blocking was carried out in 2% BSA/PBS1X for 10 min at room temperature. Cells were incubated with mouse anti‐FLAG M2 (1/200, Sigma‐Aldrich, F3165) for 45 min at RT, washed three times with dPBS1X and incubated with the secondary antibody solution containing Alexa fluo 488 (1/200, Invitrogen, A21202), Hoescht 10 μg/mL (Invitrogen, 33342) and 5 μmol/L (DOJINDO, N512‐10) Nucleolus Bright red in PBS1X for 30 min at RT. Cells were washed 3 times with dPBS1X, then coverslips were mounted on glass slides using Fluoromount G (Invitrogen, 00‐4958‐02). Fluorescence images were acquired using a CSU‐X1 spinning disk microscope equipped with 63×/1.4 Plan‐Apochromat Oil DIC (Zeiss, 420782‐9900) 0.103 μm/pixel; image acquisition was performed with ZEN blue 2.6 software.

### 2.9 FRAP Assay

HeLa cells were seeded in 8‐well μ‐slides (Ibidi, 80 826) and transfected using the calcium phosphate method with 625 ng/well of plasmids encoding WT FOXL2 or the S101D, S107A, and S107D variants. Forty‐eight hours post‐transfection, FRAP assays were performed using a CSU‐X1 spinning disk equipped with Plan‐APOCHROMAT 63×/1.4 Oil and a FRAP ILAS 2 module (Gataca) and controlled by MetaMorph software 7.10 (RRID:SCR_002368). All experiments were conducted at 37°C with 5% CO2. For each cell a small nuclear region of interest (ROI) (excluding the nucleoli) was photobleached and the fluorescence recovery was monitored over a 31‐s period with an acquisition interval of 62.5 ms between pictures. Using the EasyFRAPweb application [34], we determined the T‐half value for FOXL2 WT and the variants, corresponding to the time required to recover 50% of the maximum fluorescence after photobleaching, which is inversely related to protein mobility.

### 2.10 RNA‐Sequencing

KGN WT/WT cells stably transduced with WT FOXL2 and its variants were seeded in 6‐well plates at 25% confluence, after 16 h, cells were treated with 500 ng/mL doxycycline for 24 h. We used KGN transduced with WT FOXL2 without doxycycline induction as a control. At confluence, total RNA was extracted using the Tri Reagent (Life technologies, 15596‐018). Chloroform was added to separate RNA from DNA and proteins. RNA was precipitated by adding glycogen and isopropanol. Finally, the RNA was washed with 75% ethanol, left to dry and resuspended in water. After RNA extraction, RNA concentrations were determined using a fluorometric Qubit RNA assay (Life Technologies, Grand Island, New York, USA). The quality of the RNA (RNA integrity number) was determined on the Agilent 4200 TapeStation (Agilent Technologies, Palo Alto, CA, USA) as per the manufacturer's instructions. To construct the libraries, 350 ng of high‐quality total RNA sample (RIN = 10) was processed using Stranded mRNA Prep kit (Illumina) according to manufacturer instructions. Briefly, after purification of poly‐A containing mRNA molecules, mRNA molecules are fragmented and reverse‐ transcribed using random primers. Replacement of dTTP by dUTP during the second strand synthesis will permit to achieve the strand specificity. Addition of a single A base to the cDNA is followed by ligation of Illumina adapters. Libraries were quantified by Qubit (Invitrogen) and profiles were assessed using the DNA 1000 kit on an Agilent TapeStation 4200. Libraries were sequenced on an Illumina Nextseq 2000 instrument using 59 base‐length reads in a paired‐end mode. After sequencing, a primary analysis based on AOZAN software (ENS, Paris) [35] was applied to demultiplex and control the quality of the raw data (based of FastQC modules/version 0.11.5). Fastq files were then aligned using STAR algorithm (version 2.7.11b, RRID:SCR_004463), on the Ensembl release 113 reference (RRID:SCR_002344). Reads were then counted using RSEM (v1.3.3, RRID:SCR_000262) and the statistical analyses on the read counts were performed with R (version 4.4.2) and the DESeq2 package (DESeq2_1.42.1, RRID:SCR_000154) to determine the proportion of differentially expressed genes between two conditions. We used the standard DESeq2 normalization method in a pair‐wise fashion (DESeq2's median of ratios with the DESeq function), with a pre‐filter of reads and genes (reads uniquely mapped on the genome, or up to 10 different loci with a count adjustment, and genes with at least 10 reads in at least 3 different samples). Following the package recommendations, we used the Wald test with the contrast function and the Benjamini‐Hochberg FDR control procedure to identify the differentially expressed genes. R scripts and parameters are available on the platform, https://github.com/GENOM‐IC‐Cochin/RNA‐Seq_analysis. The two‐way hierarchical clustering based on all DEGs (displayed in Figure S5) was performed with Morpheus program (RRID:SCR_014975, from the Broad Institute) using Kendall's correlation.

Each condition consists of 3 biological replicates. To identify differentially regulated genes by the mutants compared to the WT, we performed a sequential analysis (Figure 3). In step 1, we defined the DEGs of the WT and the DEGs for each mutated (Mut) form through comparisons between the subsets corresponding to WT + DOX versus WT‐DOX conditions (with a *p*‐adj ≤ 0.05 and a Log_2_FC ≥ 0.585 or ≤ −0.585, which correspond to a FC ≥ 1.5 in either direction) and similarly for the mutated forms (Mut + DOX vs. WT‐DOX). Then in a second step, we compared the DEGs list obtained from the comparison WT + DOX versus Mut + DOX (*p*‐adj ≤ 0.05) with the union of the DEGs of the WT and the Mut (from step1) to ensure that the relevant transcripts were found to be modulated by one or the other FOXL2 forms (to increase the reliability of our analysis). After that, we selected transcripts/genes with an FC ≥ 1.5 in either direction. This comparison led to a subset of DEGs, which we classified as cases of loss‐of‐function (LOFs) and gain‐of‐function (GOFs). We also identified a set of transcripts/genes with a similar regulation by focusing on those common to the DEGs of the WT and the mutated forms (from Step 1) with a maximum FC of 1.5 in either direction (to limit the number of transcripts in the analysis which in some cases had a non‐significant *p*‐value and high FC) and not being significantly different in the Mut + DOX versus WT + DOX comparison.

### 2.11 Immunoprecipitation for MS

KGN WT/WT cells stably transduced with WT FOXL2 or its variants were cultivated in 150 cm2 flasks and treated (at 50% confluence) with 500 ng/mL doxycycline to induce transgene expression. We used KGN transduced with WT FOXL2 without doxycycline induction as a control. To distinguish endogenous from exogenous FOXL2, we used an anti‐FLAG antibody (RRID:AB_259529, Sigma‐Aldrich, F3165) targeting FOXL2‐3XFLAG. For protein extraction, cells were lyzed in ice‐cold lysis Buffer (50 mM Tris, 150 mM NaCl, 1 mM ethylenediaminetetraacetic acid (EDTA), 1% Triton X‐100, pH 7.6) supplemented with protease inhibitors (Complete mini EDTA‐free cocktail, Roche), phosphatase inhibitors (PhosSTOP, Roche). The lysates were transferred to protein Lobind tubes (Eppendorf, EP0030108116). We added 0.5 μL of M2 anti‐FLAG antibody (3.8–4.2 mg/mL) to tubes containing 50 μL of pre‐washed Dynabeads Protein G (Invitrogen, 100.04D) with rotational mixing for 10 min at ambient temperature. 1.38 mg of total protein were incubated with beads to which antibodies were attached with rotational mixing for 1 h at room temperature. After several washes, the resulting FOXL2‐3xFLAG‐protein complexes were processed for MS analysis.

### 2.12 Interactome Analysis

For sample preparation prior to LC–MS/MS analysis, beads from pulldown experiments were incubated overnight at 37°C with 20 μL of 50 mM NH4HCO3 buffer containing 1 μg of sequencing‐grade trypsin/Lys C mix (Promega). Before LC–MS/MS analysis, the digested peptides were loaded and desalted on evotips provided by Evosep (Odense, Denmark) according to the manufacturer's procedure. Analyses were performed on a timsTOF Pro HT mass spectrometer (Bruker Daltonics, Bremen, Germany) coupled to an Evosep One system (Evosep, Odense, Denmark), operated with the manufacturer's 40SPD Whisper Zoom method. This method relies on a 32.5‐min gradient and a total cycle time of 36 min. Peptide separation was carried out on a C18 analytical column (0.075 × 150 mm, 1.7 μm beads, Aurora Elite CSI, IonOpticks), maintained at 50°C and operated at 200 nL/min. Mobile phases were (A) H_2_O/0.1% FA and (B) ACN/0.1% FA (ThermoFisher Scientific). The timsTOF Pro HT was operated in DIA‐PASEF mode, based on 12 pyDiAID frames, each with three mass windows, resulting in a 0.975 s cycle time as described in Bruker Application Note LCMS 218. Collision energy was applied stepwise as a function of ion mobility. Raw MS files were processed with Spectronaut (version 20.1.250624.92449). Database searches were performed against the SwissProt *Homo sapiens* database (downloaded March 2025, 20 429 entries).

Search parameters included: 20 ppm precursor mass tolerance, 0.05 Da fragment mass tolerance, specific tryptic cleavage with up to two missed cleavages. Considered modifications were acetylation (protein N‐term), oxidation (M), and deamidation (NQ) as variable, and β‐methylthiolation (C) as fixed.

Protein identification was filtered at 1% *Q*‐value threshold at both precursor and protein levels. Quantification was carried out using the Spectronaut Quantification Module with default settings. Protein inference was performed with the IDPicker algorithm. Multivariate statistics on protein or peptide measurements were performed using Qlucore Omics Explorer 3.9 (Qlucore AB, Lund, SWEDEN). A positive threshold value of 1 was specified to enable a log2 transformation of abundance data for normalization that is, all abundance data values below the threshold will be replaced by 1 before transformation. The transformed data were finally used for statistical analysis that is, evaluation of differentially present proteins or peptides between two groups using a student's bilateral *t*‐test. A *p*‐value better than 0.01 was used to filter differential candidates. The two‐way hierarchical clustering based on all DIPs (displayed in Figure S6) was performed with Qlucore program using Kendall's correlation.

MS identified 3105 proteins in total. The abundance values for each IP sample were normalized using a Log_2_ transformation. Each condition consists of 3 biological replicates. Protein abundance values for each condition were compared using two‐tailed Student's *t*‐tests. To identify differences in direct and indirect protein–protein interactions (PPIs), we performed a sequential analysis (Figure 3). In step 1, we defined the interactomes of the WT and each mutated (Mut) form through comparisons between the subsets corresponding to WT + DOX (i.e., induced mild over‐expression) versus WT‐DOX conditions (with *p*‐value ≤ 0.01 and Log_2_FC > 0) and similarly for the mutated forms (Mut + DOX vs. WT‐DOX). Then, we compared the protein list obtained from the comparison WT + DOX versus Mut + DOX with the union of the interactomes of the WT and the Mut (from step1) to ensure that the relevant proteins were found to interact with one or the other FOXL2 forms (to increase the reliability of our analysis, step 2). This comparison led to a subset of differentially interacting proteins (DIPs, which we classified as LOIs for lost/decreased interactions and GOIs for gained/increased interactions). For the identification of LOIs and GOIs, we did not set a Log_2_FC threshold. Indeed, after applying the threshold *p*‐value ≤ 0.01 to the WT + DOX versus Mut + DOX comparison, the DIPs were predominantly distributed above an FC of 1.5 in both directions (The minimum FC values: for S101A, repressed genes: 1.45 and activated genes: 1.60; for S101D, repressed: 1.63 and activated: 1.52; for ΔC, repressed: 1.48 and activated: 1.98). Proteins with a Log_2_FC < 0 were classified as LOIs and those with a Log_2_FC > 0 as GOIs. We also identified a set of proteins with retained interactions by focusing on those common to the interactomes of the WT and the mutated forms (from Step 1) with a maximum FC of 1.5 in either direction and not being significantly different in the Mut + DOX versus WT + DOX comparison.

## 3 Results

### 3.1 Modification of Ser 101 and Ser 107 of FOXL2 Modulates Its Activity

**FIGURE 1:**
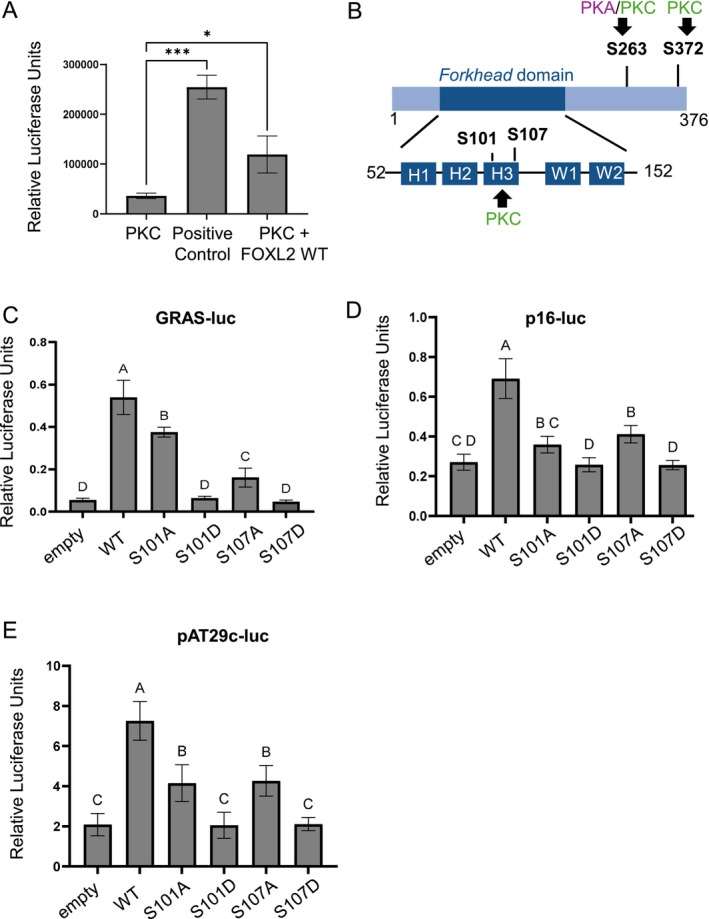
Modification of Ser 101 and Ser 107 of FOXL2 modulates its activity. (A) In vitro phosphorylation assay by PKCα. Positive control: CREBtide (KRREILSRRPSYR) PKCα substrate. Each value is representative of three biological replicates. Errors bars represent the standard deviation (SD). Statistical significance was tested using a Tukey test: *p < 0.05, ***p < 0.001. (B) Representation of FOXL2 protein with serine positions phosphorylated by PKA and PKC. (C–F) Luciferase assays in HeLa cells transfected with GRAS‐luc (C) p16‐luc and (D) pAT29C‐luc (E), respectively, with or without overexpression of WT FOXL2 or the mutated forms. The constructs used consist of promoter fragments fused to the luciferase reporter gene (either artificial promoters containing concatemerized FOXL2 response elements or natural promoters) [37, 38]. For each PKC‐phosphorylation site identified within the FOXL2 FHD, Ser residues were replaced by Ala (A) to mimic the unphosphorylated state and by aspartate (D) to mimic the phosphorylated state. Each value is representative of six biological replicates. Errors bars represent the standard deviation (SD). Letters A–D denote Tukey multiple‐comparison groupings; groups that do not share a letter are significantly different.

In a previous study, we demonstrated that FOXL2 undergoes extensive PTM, including phosphorylation, following its transient over‐expression in COS‐7 cells [11]. To further investigate the phosphorylation of FOXL2 by specific PKs, we performed in vitro phosphorylation assays using purified (bacterially‐expressed) FOXL2 [36] and various recombinant PKs (namely, AKT1, PKA, PKCα and GSK3β). Phosphorylation was assessed by coupling the kinase assay to an ADP detection system, enabling the quantification of enzymatic activity via luminescence measurements. Such assays showed that PKC was able to phosphorylate FOXL2 in our experimental conditions (Figure 1A). The analysis of intact FOXL2 by MS showed mass shifts of +80 Da and +160 Da, suggesting the existence of two phosphorylation states (i.e., mono‐ and diphosphorylation) (Figure S1). Upon digestion of FOXL2 with trypsin and chymotrypsin, we identified four FOXL2 phosphorylated sites (Figure S2). These results suggest that FOXL2 can only be phosphorylated on one or two of these residues simultaneously in our experimental conditions. Two of these sites, S101 and S107, are located within the third helix (H3) of the FHD. The remaining two sites, S263 and S372, are located in the C‐terminal domain of FOXL2 (Figure 1B). To investigate the functional relevance of phosphorylation at these positions, site‐directed mutagenesis was performed to replace the relevant Ser residues with alanine (A), a non‐phosphorylatable amino acid structurally close to Ser, and with aspartate (D), a residue that mimics the negative charge of phosphorylated Ser at the intracellular pH (i.e., phosphomimetic). We then assessed the activity of these mutants by evaluating their transactivation ability on reporter promoter known to be direct targets of FOXL2 (namely, *p16/INK4a*) [6, 39]. In addition, FOXL2 activity was tested on two reporters driven by artificial promoters: pAT29C, which contains two tandem FOXL2‐response elements (FLRE) [37] and 3 × GRAS, consisting of three repeats of an AP‐1/SMAD3/FOXL2 composite response element identified in the murine *GnRH* receptor promoter [40]. Mutants S263A/D and S372A/D exhibited less consistent and pronounced differences relative to WT FOXL2 than S101A/D and S107A/D and we therefore focused on the latter two sets of variants for further analyses (Figure S3A–C). In contrast, S101A and S107A resulted in a reduced transcriptional activity and, more strikingly, substitutions S101D and S107D led to a complete loss of activity on these promoters (Figure 1C–E). The latter finding shows that the S → D substitution leads to profound alterations of FOXL2 transcriptional activity.

**FIGURE 2:**
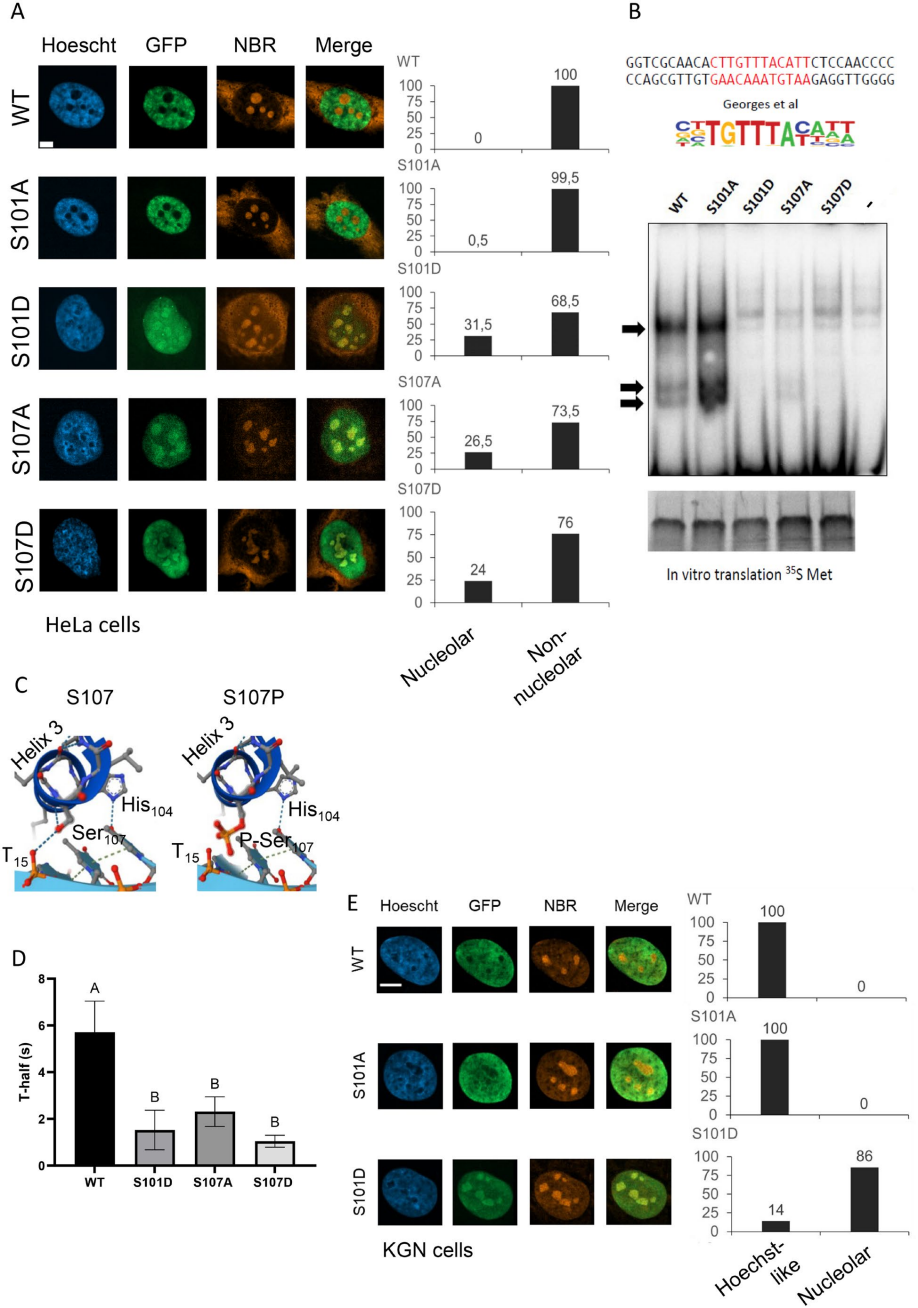
Modification of Ser 101 and Ser 107 leads to a loss of FOXL2 interaction with DNA and its enrichment in the nucleoli. (A) Nucleolar distribution of FOXL2 in HeLa cells overexpressing GFP‐tagged FOXL2 (WT or the indicated mutants). Left‐most panel: Nuclear stained with Hoechst 33342. Second panel: FOXL2 GFP variants. Third panel: Nucleolar staining with Nucleolus Bright Red (NBR) (N512‐10, DOJINDO). Right‐most panel: Merged images of FOXL2 variants with nucleolar staining. Scale bar: 5 μm. The graphs on the right display the quantitative distribution of FOXL2 variants across subnuclear compartments. Percentages were calculated from at least 200 transfected HeLa cells. (B) EMSA. Upper panel: Probe derived from the forkhead consensus motif [6]. Lower panel: EMSA for the relevant variants carried out with in vitro synthesized proteins. (‐): empty vector control (C) AlphaFold simulations for FOXL2 with Ser107 in both phosphorylated and unphosphorylated states (D) Quantitative analysis of FRAP experiments carried out in HeLa cells transiently expressing GFP‐fused WT FOXL2 or the mutated forms, except for S101A which does not localize in the nucleolus. The time required for the recovery of half of the maximum fluorescence after photobleaching (t_1/2_, in seconds) was determined from the recovery curves. Letters A and B refer to statistical categories in a Tukey test. WT, *n* = 5; S101D, *n* = 4; S107A, *n* = 5; S107D, *n* = 2. We acknowledge that the statistical power for the S107D group is limited due to sample size *n* = 2. (E) Immunofluorescence displaying the subcellular localization of WT FOXL2 and the mutated forms fused to 3xFLAG in stably transduced KGN cells. Scale bar: 5 μm. The graphs on the right display the quantitative distribution of FOXL2 variants across subnuclear compartments. Percentages were calculated over 200 transduced KGN.

Many FOXL2 variants have been shown to induce protein aggregation [41]. To assess whether this was the case for S101A/D and S107A/D, we expressed the mutated proteins fused to the green fluorescent protein (GFP) in HeLa cells. No obvious aggregation was observed using epifluorescence microscopy. We also assessed FOXL2 mobility using Fluorescence Recovery After Photobleaching (FRAP). All tested mutants recovered fluorescence in less than 2 min 5 s, indicating that they did not aggregate (Figure S4). Variant S101A displayed a localization comparable to that of WT FOXL2 (Figure 2A), whereas S101D, S107A, and S107D mutants displayed a high proportion of cells (24%–31.5%) exhibiting a seemingly nucleolar localization. After excluding a potential aggregation of the mutants, we hypothesized that the lack of activity was linked to a decreased ability to interact with DNA. Indeed, it is known that phosphorylation of the 101 and 107 orthologous positions in FOXO1, in combination with two other residues not present in FOXL2, alters FOXO1 interaction with DNA [42]. To assess the DNA‐binding capacity of FOXL2 mutants, we performed an electrophoretic mobility shift assay (EMSA) (Figure 2B). The DNA probe used contained a consensus motif corresponding to a sequence we had previously identified [43]. WT and mutated FOXL2 proteins were generated by in vitro transcription and translation. WT FOXL2 efficiently bound the DNA probe, as shown by a clear mobility shift. The lower mobility shifts observed were consistent with DNA binding by monomeric FOXL2, while higher molecular weight bands suggested the formation of higher‐order FOXL2‐DNA complexes [44]. The S101A mutant also bound the probe, producing a band of greater intensity than that of the WT, which may reflect a stronger interaction with DNA. In contrast, no shift was detected for the S101D and S107D mutants, indicating a complete loss of DNA‐binding capacity. The S107A mutant produced a faint band, consistent with a weaker binding to the probe. These results indicate that the S107A, S101D, and S107D mutants have partially or completely lost their ability to bind the forkhead consensus sequence, which correlates with the results of the luciferase assays.

Following these experiments, we performed a simulation on AlphaFold for FOXL2 with Ser107 in both phosphorylated and unphosphorylated states (Figure 2C). The DNA probe used is the same as that used for EMSA, namely the consensus sequence for forkhead factor binding. This simulation clearly shows a hydrogen bond between the unphosphorylated S107 residue and position T15 of the DNA sequence, a bond that is lost when the S107 residue is phosphorylated. This is consistent with the results obtained by EMSA and luciferase assays.

### 3.2 Nucleolar Enrichment and Rapid Dynamics of FOXL2 S101/S107 Mutants

As mentioned above, mutants S101D, S107A, and S107D result in a high proportion of transfected cells exhibiting subnuclear structures characterized by elevated local concentrations of GFP‐fused proteins. The structures in which the S101D, S107A, and S107D FOXL2 mutants accumulated were morphologically consistent with nucleoli. To verify this, we transfected HeLa cells with constructs driving the expression of WT and mutated FOXL2 forms and subsequently labeled the nucleoli with Nucleolus Bright Red, an rRNA marker. As expected, we observed a clear colocalization of both fluorescent signals (Figure 2A).

We also examined the mobility of the FOXL2 mutants in more details using FRAP in transiently transfected HeLa cells. Fluorescence recovery of a small region of interest was monitored over 19 s for each mutant displaying nucleolar localization. After calculating the *t*_1/2_ (i.e., time required to recover 50% of the maximum fluorescence after photobleaching), which is inversely related to protein mobility, we found that WT FOXL2 displayed a lower mobility compared to the mutants enriched in the nucleolus (Figure 2D). We did not observe any significant differences for the other mutants. The higher mobility of the mutants relative to WT FOXL2 is consistent with a reduction, or a complete loss, of their interaction with DNA, which would facilitate a faster diffusion.

To verify that the nucleolar enrichment observed for some mutants was not cell‐line specific, we performed an immunofluorescence in KGN cells transduced with FOXL2‐WT‐3XFLAG or the relevant mutants, using an antibody recognizing the 3XFLAG tag to distinguish “exogenous” FOXL2 from the endogenous protein. These KGN cells used had been previously modified using CRISPR/Cas9 to carry two WT alleles of FOXL2 with a low endogenous expression [45]. For further experiments, mutants S101A and S101D were selected as representative of those that display differential nucleolar localization. Cells expressing S101A displayed a subcellular localization pattern comparable to that of WT FOXL2 (Figure 2E). In contrast, the localization of the S101D mutant was consistent with that observed in HeLa cells. Notably, nearly all S101D‐expressing cells exhibited a nucleolar enrichment.

### 3.3 Phospho‐Mimetic Substitution S101D and C‐Terminal Loss Affects FOXL2‐Dependent Transcription in KGN Cells

Next, we studied the transcriptomic effects of the point mutations (i.e., S101A and S101D) and the C‐terminal truncation (ΔC, lacking a segment from 153 to 376 residues) of FOXL2 in transduced KGN cells using RNA‐seq. For this, we compared the transcriptome of cells expressing WT FOXL2 with that of the different variants. According to RNA‐seq data, FOXL2 variants were overexpressed about 3.95 ± 0.39 times with respect to the endogenous gene.

A hierarchical clustering (see heatmap in Figure S5 and volcano plots in Figure S6) based on all DEGs revealed that S101A clusters with the WT, suggesting a low functional impact of the mutation. On the contrary, the S101D and ΔC mutants clustered with the control condition (i.e., without induction).

**FIGURE 3:**
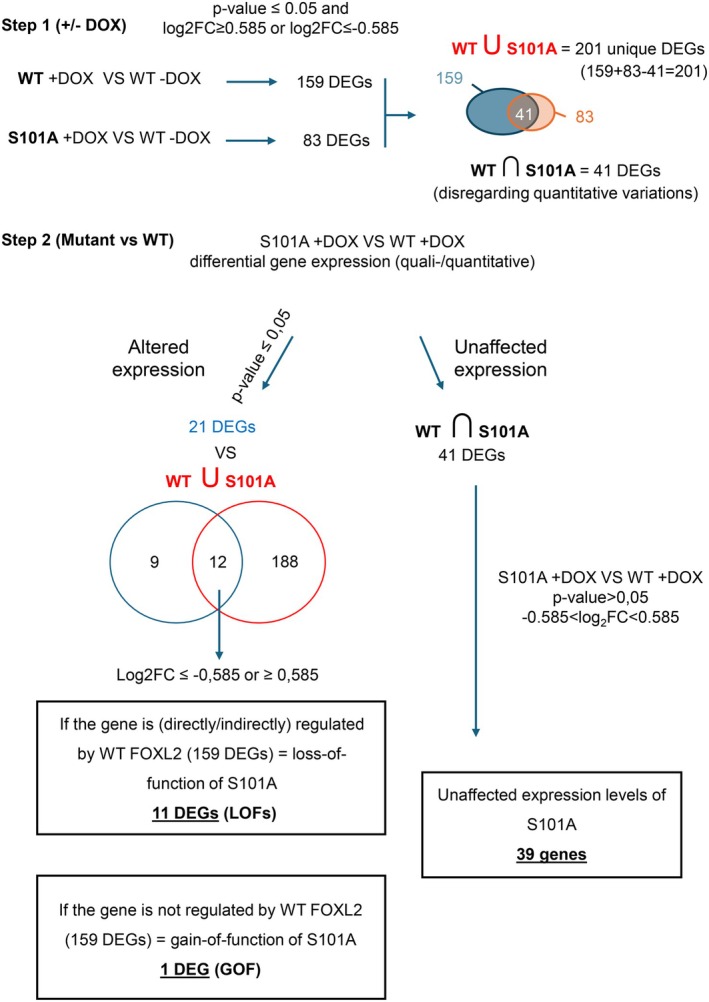
Flowchart of the analysis of the transcriptomic impact of the FOXL2 mutated forms. The analysis of DEGs of the S101A variant was taken as an example. This method was applied to the other variants in the transcriptomic analysis and in the PPI analysis. Step 1: definition of the DEGs subset of the WT and the mutated forms. Symbols U and ∩ stand for the union and intersection of the relevant subsets (DEGs subsets), respectively. Step 2: left panel: identifying DEGs between S101A (+DOX) versus WT (+DOX). A comparison between this subset (22 DEGs) with the union of the DEGs of the WT and the S101A (from step 1) ensures that the relevant transcripts were found to be modulated by one or the other FOXL2 forms increasing the reliability of our analysis. LOFs: loss‐of‐function, GOFs: gain‐of‐function. Right panel: to identify retained regulations we focused on the transcripts/genes common to the DEGs of the WT and S101A with a maximum FC of 1.5 (arbitrary threshold) in either direction and not being significantly different in the S101A + DOX versus WT + DOX comparison.

To identify differentially regulated genes by the mutants compared to the WT, we performed a sequential analysis (Figure 3). In step 1, we defined the differentially expressed genes (DEGs) of the WT and for each mutated (Mut) condition through comparisons between the transcriptome of cells stably transduced with the WT form with and without doxycycline induction (WT + DOX vs. WT‐DOX conditions) and similarly for the mutated forms (Mut + DOX vs. WT‐DOX). In these comparisons, we focused on DEGs with an adjusted *p* value (*p*‐adj) ≤ 0.05 that had, in addition, a log_2_‐fold change (Log_2_FC) ≥ 0.585 or ≤ −0.585, which correspond to a FC ≥ 1.5 in either direction. In a second step, we compared the DEG list obtained from the comparison WT + DOX versus Mut + DOX (*p*‐adj ≤ 0.05) with the union of the DEGs of the WT and the Mut (from step1) to ensure that the relevant transcripts were found to be modulated by one and/or the other FOXL2 forms (to increase the reliability of our analysis). This comparison led to a subset of DEGs, which we classified as cases of LOFs, as expected, and gain‐of‐function (GOFs). We also identified a set of transcripts/genes with a similar regulation by focusing on those common to the DEGs of the WT and the mutated forms (from Step 1) with a maximum FC of 1.5 in either direction (to limit the number of transcripts in the analysis which in some cases had a non‐significant *p*‐value and high FC) and not being significantly different in the Mut + DOX versus WT + DOX comparison.

**FIGURE 4:**
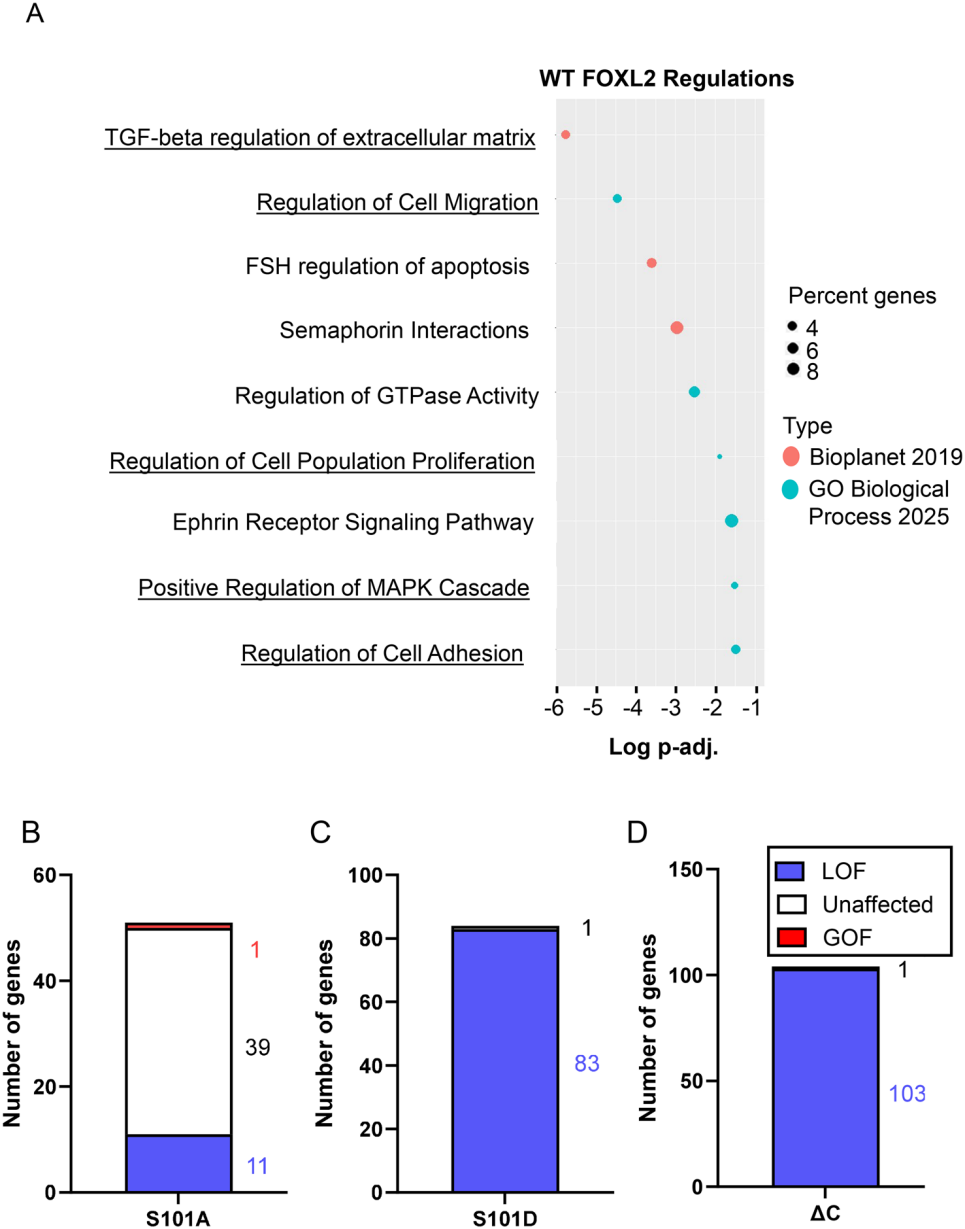
Phospho‐mimetic S101D and C‐terminal loss rewire FOXL2‐dependent transcription in KGN cells. (A) Bubble plot of the GSEA of the 159 genes directly or indirectly regulated by WT FOXL2 (comparison of WT + DOX vs. WT − DOX). The underlined biological processes were also enriched in primary murine granulosa cells according to [45]. (B–D) Graphical representation of DEGs (LOFs and GOFs) and genes with unaffected expression levels in S101A, S101D and ΔC mutants.

For WT FOXL2, we identified 159 DEGs (WT + DOX vs. WT‐DOX), corresponding to genes directly or indirectly modulated by WT FOXL2 (Table S1). Among them, 65 were downregulated and 94 upregulated. A gene set enrichment analysis (GSEA) using Enrichr [45, 46, 47, 48] of all DEGs demonstrated substantial similarities with the broad range of functional categories previously identified in primary mouse granulosa cells [45] (Figure 4A).

**FIGURE 5:**
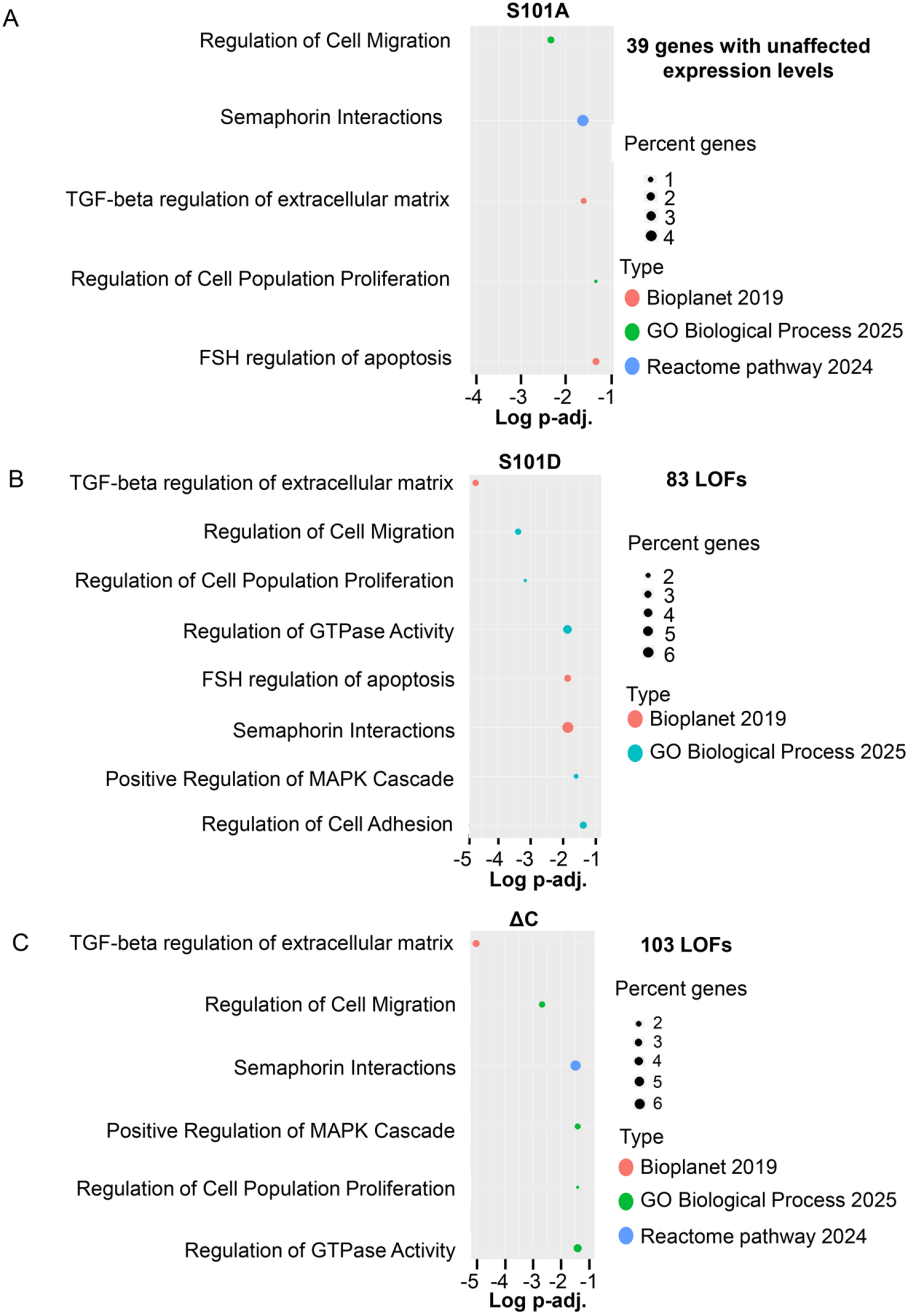
Transcriptomic impact of the S101D modification and C‐terminal truncation (ΔC) in KGN cells. (A) Bubble plot of the GSEA of the 39 genes with unaffected expression levels in S101A (comparison of S101A + DOX vs. WT + DOX). (B) Bubble plot of the GSEA of the 83 genes for which there was a LOF in S101D (comparison of S101D + DOX vs. WT + DOX). (C) Bubble plot of the GSEA of the 103 genes for which there was a LOF in ΔC (comparison of ΔC + DOX vs. WT + DOX).

Figure 4B–D shows the DEGs by FOXL2 variants and genes with conserved expression levels. The S101A variant had only 11 LOFs (upregulated/downregulated by WT and downregulated/upregulated in the comparison S101A + DOX/WT + DOX), 1 potential GOF, and 39 genes with conserved expression (Figure 4B and Table S2). A GSEA of the latter revealed substantial similarities with the biological process found to be regulated by the WT in this study (Figure 5A). We conclude that S101A basically behaves like WT FOXL2. In turn, the S101D variant displayed 83 LOFs (Figure 4C and Table S3), whereas the ΔC‐terminal variant had 103 LOFs (Figure 4D and Table S4). For these two mutated forms, we observed no genes with similar expression levels as those found in the WT condition, indicating a total loss of transcriptional activity. This result is consistent with the EMSA experiment, which demonstrated the inability of S101D to bind DNA. These results confirm that direct DNA binding is required for proper gene regulation (S101D) (no indirect effects via PPIs) and the essential role of the C‐terminal domain for proper target regulation.

A GSEA of all of the S101D and the ΔC LOFs (separately) showed enrichments in similar functions (Figure 5B,C). A comparison of the GSEA of the LOFs of these two variants with the enriched process for WT FOXL2 revealed a “shut‐down” of the processes regulated by the latter.

### 3.4 Mutation of FOXL2 Alters Its Interactions With Its Partners

To understand the effects of S101 phosphorylation and C‐terminal truncation on direct and indirect PPIs, we performed an interactome analysis of WT, S101A, S101D, and ΔC by immunoprecipitating FOXL2 followed by MS in transduced KGN cells.

Protein abundance profiles were compared between mutants and WT FOXL2 to assess the impact of these modifications on their interactome. A hierarchical clustering (see heatmap in Figure S7) based on all differentially interacting proteins (DIPs) revealed, as expected, the clustering of the replicates. While S101A and ΔC variants showed moderate deviations from WT FOXL2, the S101D mutant was distinctly separated, suggesting a significant shift in the PPIs of the latter.

To identify differences in direct and indirect PPIs, we performed a sequential analysis in the same way as for the transcriptomic analysis (Figure 3). However, we used different thresholds and other criteria for detecting lost/decreased interactions (LOIs) and gained/increased interactions (GOIs). Specifically, the DIPs were defined using a *p*‐value ≤ 0.01 and for a Log_2_FC > 0. For the identification of LOIs and GOIs, we did not set a particular Log_2_FC threshold because after applying the *p*‐value ≤ 0.01 threshold to the WT + DOX versus Mut + DOX comparison, the DIPs were predominantly distributed above an FC of 1.5 in both directions (see details in the materials and methods section and Table S5). Proteins with a Log_2_FC < 0 were classified as LOIs and those with a Log_2_FC > 0 as GOIs.

**FIGURE 6:**
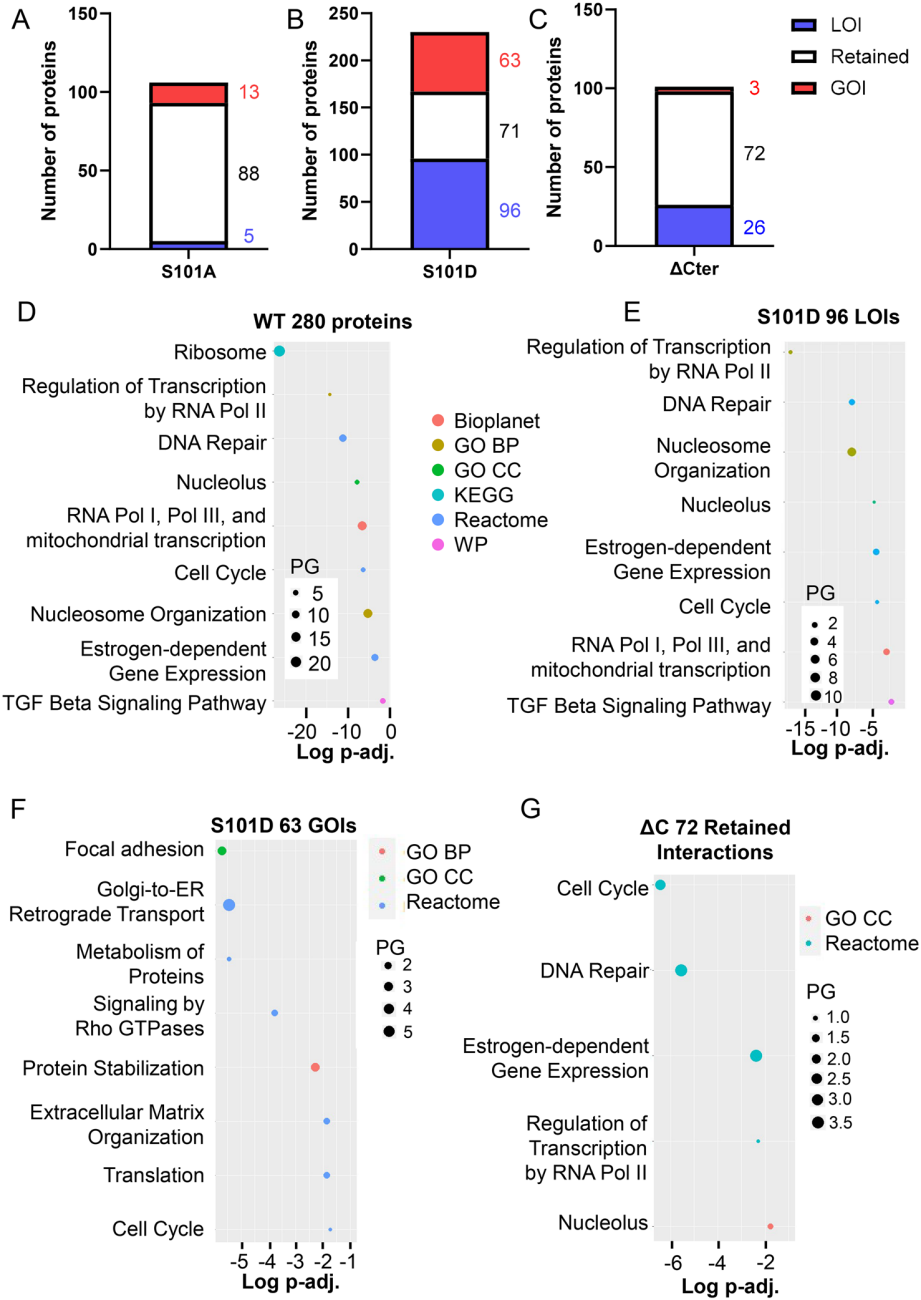
Mutation of FOXL2 alters its interactions with its partners. (A–C) Graphical representation of differentially interacting proteins (DIPs: LOIs and GOIs) and retained interactions in S101A, S101D and ΔC mutants. (D) Bubble plot of the enrichment analysis of the 280 proteins of the WT FOXL2 interactome (direct and indirect interactions, comparison of WT + DOX vs. WT − DOX). (E) Bubble plot of the enrichment analysis of the 96 LOIs in the FOXL2 S101D mutant (comparison of S101D + DOX vs. WT + DOX). (F) Bubble plot of the enrichment analysis of the 63 GOIs of the FOXL2 S101D mutant (comparison of S101D + DOX vs. WT + DOX). (G) Bubble plot of the enrichment analysis of the 72 proteins for which there was a conservation of interaction in the ΔC. Note that ΔC retains most of WT FOXL2 interactions that are lost by S101D (comparison of ΔC + DOX vs. WT + DOX). PG: Percent gene; Bioplanet: Bioplanet 2019; GO BP: GO Biological Process 2025; GO CC: GO Cellular Component 2025; KEGG: KEGG 2021 Human; Reactome: Reactome pathways 2024; WP: Wikipathways 2024 Human.

Figure 6A–C shows the DIPs and the conserved interactions of the FOXL2 variants. The S101A variant had 5 LOIs, 13 GOIs, and 88 retained interactions (Figure 6A and Table S6). The S101D variant had 96 LOI, 63 GOI and 71 retained interactions (Figure 6B and Table S7). The ΔC‐terminal variant had 26 LOI, 3 GOI and 72 retained interactions (Figure 6C and Table S8). Given the scarcity of DIPs for S101A and the absence of enrichment in specific biological pathways, we focused on the other variants in the following analysis.

To identify signaling pathways and functions involving FOXL2 and its direct and indirect partners, we analyzed the 280 proteins in the WT interactome using Enrichr (Figure 6D). This analysis confirmed our previous observations [49], showing in a different cell type the potential involvement of FOXL2 and its partners in the regulation of DNA replication and repair, the cell cycle, ribosome biogenesis, and chromatin remodeling. This analysis also revealed a significant enrichment of nucleolar proteins. We further compared the 280 partners of WT FOXL2 with a list of nucleolar proteins previously identified [50] and found 39 proteins in common. Among them, there was a high proportion of ribosomal proteins (RPs), but also nucleolin, a major nucleolar component [51]. We also detected MACROH2A1, a histone variant present at the promoters of methylated rDNA genes [52]. WT FOXL2 was also found to interact with UBTF, essential for the activation of rDNA gene transcription [53].

The S101D mutation markedly affected the FOXL2 interactome, with 96 LOIs, accounting for over one‐third of the interactors identified in the WT condition (Figure 6B). Most of the LOIs concerned proteins involved in biological process like transcription by RNA polymerase II, such as many TFs (e.g., JUN, RUNX1, TEAD1/3, USF1/2, etc.) and proteins involved in chromatin remodeling (e.g., HLTF, MECP2, SMARCA5, etc.) (Figure 6E). The S101D mutant also exhibited LOIs with proteins involved in the transcription by RNA polymerases I and III. They include, notably, UBTF, which is essential for rRNA transcription [54], as well as four members of the nuclear factor I (NFI) family NFIA, NFIB, NFIC, and NFIX, which cooperate with TFIIIC to facilitate RNA pol III transcription termination [55]. Additionally, reduced interactions were observed with proteins involved in other biological processes, including DNA replication/cell cycle regulation (e.g., RPA1/2/3, CENPC, TOP2A…) and DNA repair. Two of the proteins involved in the latter process are XRCC5 and 6, which mediate DNA double‐strand break repair by non‐homologous end‐joining. Interactions WT‐FOXL2‐XRCC6 have previously been reported by us and others [38, 49, 56]. In contrast, 71 PPIs (direct and indirect) were retained by the S101D mutant, notably with 24 RPs. Although, approximately half of the nucleolar proteins identified in the WT FOXL2 interactome (according to the dataset described in [50]) were lost by S101D, it retained interactions with GTF3C1, GTF3C2, and GTF3C3, which are components of the TFIIIC complex [57]. This mutant displayed 63 potential GOIs (Figure 6B). Their functional enrichment analysis revealed an overrepresentation of proteins involved in process such as focal adhesion, Rho GTPase signaling and many others (Figure 6F). We also detected novel interactions with RPs. By comparing the list of novel interactors of the S101D mutant with the nucleolar proteome [50], we identified five nucleolar proteins that interact with S101D.

Rather surprisingly, the ΔC variant exhibited only a minimal LOI, with only 26 interactors of WT FOXL2 no longer detected (e.g., RPL2/13/24/28 and GTF3C1 and GTF3C3 (Figure 6C)). As displayed in Figure 6G, most biological processes enriched in the WT FOXL2 interactome remained enriched for ΔC. As expected, the comparison of enriched biological processes between S101D and ΔC revealed that most of them were lost by S101D.

## 4 Discussion

Here, we show that purified PKC is able to phosphorylate FOXL2 in vitro at S101/S107, mapping to H3 of the FHD. Our subsequent data using phosphomimetic mutants suggest that phosphorylation of S101 and, by extension, of S107 is expected to act as a molecular switch that tunes DNA binding, gene regulation, partner selection, and subnuclear partitioning. Indeed, the substitution S101D was found to abolish DNA binding in vitro, leading to a collapse of promoter activity on multiple FOXL2‐responsive promoters in luciferase assays, while the conservative substitution S101A was largely neutral.

Interestingly, we found that S101D and to a lesser extent the S107D/S107A variants relocalized to nucleoli without evidence of aggregation in HeLa cells, and this was recapitulated in KGN cells. This suggests that phosphorylation near or at the DNA‐binding helix reduces FOXL2–DNA affinity, which would in turn favor nucleolar residence. These effects are mirrored by the results of our transcriptomic and proteomic studies. The S101D variant displayed a massive LOF at the transcriptomic level. As noted above, this outcome is expected to result from a DNA binding impairment as showed by the EMSA experiment. This is consistent with the fact that S101D loses interactions with dozens of Pol II–associated TFs (such as FOXC1, DRAP1, HLTF), chromatin remodeling factors (including, e.g., SUPT16H, SSRP1, RSF1), Pol I/III regulators such as UBTF and TFIIIC components and DNA repair players (XRCC6, RPA complex). The interactomic changes (LOIs) observed can be explained if many FOXL2 PPIs occur or are stabilized in a DNA‐bound state. When H3 is electrostatically perturbed (the presence of the negatively charged Asp or phosphorylation), DNA residence time decreases making large portions of the FOXL2 regulome to collapse. According to PPIs, S101D gains partners involved in Rho GTPase signaling (e.g., MAP2K1), suggesting that when DNA binding is compromised, FOXL2 shifts toward other partners and signaling nodes rather than executing its expected transcriptional program.

The ΔC variant displayed strikingly similar transcriptomic effects as those of S101D. This suggests that an intact C‐terminus is required to fully assemble productive transcriptional complexes. Contrasting with the transcriptomic findings, ΔC retained a broad set of “WT PPIs” (e.g., most Pol II–related interactions) and preferentially lost contacts with RPs and TFIIIC subunits. This also contrast with the proteomic results of S101D and reinforces the idea that many FOXL2 PPIs mediated either by the N‐terminal segment or the FHD do occur in a DNA‐bound state.

Our results suggest that the FHD, especially via its DNA recognition helix, facilitates a large subset of PPI events whereas the C‐terminus would (i) fine‐tune promoter selectivity probably through the presence of intrinsically disordered regions known to be involved in this process [58] and/or (ii) contain a domain mediating transactivation/transrepression. The apparent complementarity observed between S101D, which loses many DNA/Pol II contacts but keeps more ribosomal ones, and ΔC, which retains many Pol II contacts but loses ribosomal ones, supports a functional partition yet to be explored.

FOXL2 seems to engage in interactions with numerous nucleolar partners (e.g., many RPs, NCL, etc.) involved in ribosome biogenesis that requires exploration. S101D drops roughly half of these nucleolar interactions yet it gains/increases interactions with other nucleolar factors. Among these proteins is SDAD1, a chaperone involved in the folding of the 5S ribonucleoprotein, as well as in the assembly and nuclear export of the 60S ribosomal subunit [59]. Another identified nucleolar interactor was the methyltransferase SETD7 [60], which could promote or stabilize nucleolar residency. Interestingly, ΔC shows a rather opposite pattern for several RP interactions, pointing to the existence of FHD‐mediated versus C‐terminal–mediated interaction interfaces. The dynamic nucleolar enrichment of S101D (and S107A/D) can be explained by a reduced DNA affinity, which would free FOXL2 that would be able to diffuse and become available to be captured by nucleolar interactors.

FOXL2 is a dosage‐sensitive regulator of ovarian homeostasis. The S101D LOF at the transcriptomic level affects pathways central to granulosa cell physiology and individual genes with recognized ovarian roles (e.g., *STAR, INHBA, FST*). We hypothesize that PKC activation would lead to phosphorylation of S101 (and S107), reducing the affinity of FOXL2 for DNA therefore disrupting PPIs taking place in a DNA/chromatin‐bound state. Freed from chromatin, FOXL2 would engage in interactions with other partners, including nucleolar ones, increasing its residency in this subcompartment. This would provide a fast, reversible mechanism to silence portions of the FOXL2 transcriptional program without requiring protein degradation. The C‐terminus is proposed to contribute to promoter selectivity, gene transactivation/repression, and would also stabilize interactions with ribosomal components. Its deletion phenocopies most of S101D LOFs yet preserves most WT PPIs. C‐terminal interactions would be required, not for DNA binding per se, but to recruit/coordinate co‐factors that interface with transcription and RNA metabolism.

On more general grounds, the DNA‐recognition helix is a structural feature conserved across all FOX proteins. Our findings suggest that phosphorylation within this helix may be a conserved mechanism for rapidly toggling FOX factors between DNA‐bound and unbound states, affecting both their interactomes and their transcriptomic effects.

A strength of this study is the consistency between pathways altered in RNA‐seq and the differences observed in the interactomes. However, it also presents several limitations; we are conscious that Asp mimics charge but not the geometry of a phosphate. The study of direct PKC phosphorylation of FOXL2 at S101/S107 in the cell is also a necessary step. Although our data indicate that the S101D mutation, and by extension potential phosphorylation at this residue, reduces FOXL2 transcriptional activity and promotes its redistribution to the nucleolus, the physiological context and biological consequences of this phenomenon remain unclear. In particular, the upstream signals and PKC isoform involved, the function of nucleolar FOXL2, and its potential effects on cell growth, ribosome biogenesis, and protein translation warrant further investigation. We also note that even mild FOXL2 overexpression could potentially bias our results, so more refined experiments via a CRISPR knock‐in strategy targeting the endogenous locus are required. That said, we propose that PKC is a strong candidate to modulate the interactome of FOXL2 and the gene expression program(s) that it regulates.

## Author Contributions

Conceptualization: R.A.V., A.‐L.T. Formal analysis: L.M., R.A.V., A.‐L.T. Funding acquisition: R.A.V., A.‐L.T. Investigation: L.M., D.C., F.P., B.L., R.A.V., A.‐L.T. Methodology: L.M., R.A.V., A.‐L.T. Project administration: R.A.V., A.‐L.T. Supervision: R.A.V., A.‐L.T. Validation: R.A.V., A.‐L.T. Visualization: L.M., D.C., F.P. Writing – original draft: L.M., R.A.V., A.‐L.T.

## Funding

This work was supported by CNRS|Institut des sciences biologiques (INSB), Université Paris Cité (University Paris City), Agence Nationale de la Recherche (ANR) (ANR‐21‐CE14‐0061).

## Conflicts of Interest

The authors declare no conflicts of interest.

## Supporting information


**Figure S1:** Intact mass analysis of FOXL2 reveals phosphorylated proteoforms. Deconvoluted intact mass spectrum of recombinant FOXL2 analyzed by TIMS‐TOF mass spectrometry. Peak intensities are reported as relative signal abundance after deconvolution of the charge‐state distribution. The experimental mass of the unmodified protein was measured at 38 768.6 Da, in agreement with the theoretical mass of 38 765.6 Da (ΔM = 2.97 Da). Additional proteoforms were detected with mass increases consistent with the presence of one (+80 Da) and two (+160 Da) phosphorylation events, yielding experimental masses of 38 848.7 Da and 38 930.3 Da, respectively. The observed mass shifts correspond to the addition of phosphate groups (+79.97 Da each), confirming the occurrence of multiple phosphorylation states of FOXL2 by PKC in vitro.
**Figure S2:** Post‐translational modification mapping of FOXL2 obtained by multi‐enzymatic digestion and mass spectrometry for PKC. FOXL2 was digested using trypsin and chymotrypsin. The resulting peptides were analyzed by LC–MS/MS. Blue bars represent the identified peptides mapped onto the FOXL2 amino acid sequence. Detected post‐translational modifications are indicated above the sequence. The Serine phosphorylated by PKC are highlighted with red rectangles.
**Figure S3:** Luciferase assays in HeLa cells transfected with (A) GRAS‐luc, (B) p16‐luc and (C) pAT29C‐luc, respectively, with or without overexpression of WT FOXL2 or the mutated forms. For each PKC‐phosphorylation site identified within the FOXL2 forkhead domain, serine residues were replaced by alanine (A) to mimic the unphosphorylated state and by aspartate (D) to mimic the phosphorylated state. Each value is representative of six biological replicates. Errors bars represent SD. Letters A, B, C, D, E and F refer to statistical categories in a Tukey test.
**Figure S4:** All mutants are mobile. HeLa cells were transiently transfected with constructs expressing WT FOXL2 or FOXL2 variants fused to GFP. Fluorescence recovery after photobleaching (FRAP) was performed, and the fluorescence recovery was assessed for 2 min and 5 s. The first panel corresponds to cells before bleaching, the second to cells after bleaching of half the nucleus, and the last to fluorescence recovery after 2 min and 5 s.

**Table S1:** DE genes in WT + Dox versus WT − Dox (RNAseq analysis) and GSEA.
**Table S2:** DE genes in S101A + Dox versus WT + Dox (RNAseq analysis) and GSEA.
**Table S3:** DE genes in S101D + Dox versus WT + Dox (RNAseq analysis) and GSEA.
**Table S4:** DE genes in ΔC + Dox versus WT + Dox (RNAseq analysis) and GSEA.
**Table S5:** FOXL2 WT interactome (MS analysis) and GSEA.
**Table S6:** DIPs in S101A versus WT (MS analysis) and GSEA.
**Table S7:** DIPs in S101D versus WT (MS analysis) and GSEA for LOI and GOI.
**Table S8:** DIPs in ΔC versus WT (MS analysis) and GSEA.

## Data Availability

The RNAseq data underlying this article are available in GEO at https://www.ncbi.nlm.nih.gov/geo/, and can be accessed with accession GSE309889. The MS data underlying this article are available in PRIDE at https://www.ebi.ac.uk/pride/, and can be accessed with accession PXD076161.

## References

[1] K. L. Clark , E. D. Halay , E. Lai , and S. K. Burley , “Co‐Crystal Structure of the HNF‐3/Fork Head DNA‐Recognition Motif Resembles Histone H5,” Nature 364, no. 6436 (1993): 412–420, 10.1038/364412a0. 8332212

[2] J. Cocquet , E. Pailhoux , F. Jaubert , et al., “Evolution and Expression of FOXL2,” Journal of Medical Genetics 39, no. 12 (2002): 916–921, 10.1136/jmg.39.12.916. 12471206 PMC1757225

[3] B. S. Ellsworth , N. Egashira , J. L. Haller , et al., “FOXL2 in the Pituitary: Molecular, Genetic, and Developmental Analysis,” Molecular Endocrinology 20, no. 11 (2006): 2796–2805, 10.1210/me.2005-0303. 16840539

[4] E. De Baere , D. Beysen , C. Oley , et al., “FOXL2 and BPES: Mutational Hotspots, Phenotypic Variability, and Revision of the Genotype‐Phenotype Correlation,” American Journal of Human Genetics 72, no. 2 (2003): 478–487, 10.1086/346118. 12529855 PMC379240

[5] S. P. Shah , M. Köbel , J. Senz , et al., “Mutation of FOXL2 in Granulosa‐Cell Tumors of the Ovary,” New England Journal of Medicine 360, no. 26 (2009): 2719–2729, 10.1056/NEJMoa0902542. 19516027

[6] B. A. Benayoun , A. B. Georges , D. L'Hôte , et al., “Transcription Factor FOXL2 Protects Granulosa Cells From Stress and Delays Cell Cycle: Role of Its Regulation by the SIRT1 Deacetylase,” Human Molecular Genetics 20, no. 9 (2011): 1673–1686, 10.1093/hmg/ddr042. 21289058

[7] J. H. Kim , S. Yoon , M. Park , et al., “Differential Apoptotic Activities of Wild‐Type FOXL2 and the Adult‐Type Granulosa Cell Tumor‐Associated Mutant FOXL2 (C134W),” Oncogene 30, no. 14 (2011): 1653–1663, 10.1038/onc.2010.541. 21119601

[8] M. Uda , C. Ottolenghi , L. Crisponi , et al., “Foxl2 Disruption Causes Mouse Ovarian Failure by Pervasive Blockage of Follicle Development,” Human Molecular Genetics 13, no. 11 (2004): 1171–1181, 10.1093/hmg/ddh124. 15056605

[9] D. Schmidt , C. E. Ovitt , K. Anlag , et al., “The Murine Winged‐Helix Transcription Factor Foxl2 Is Required for Granulosa Cell Differentiation and Ovary Maintenance,” Development 131, no. 4 (2004): 933–942, 10.1242/dev.00969. 14736745

[10] N. H. Uhlenhaut , S. Jakob , K. Anlag , et al., “Somatic Sex Reprogramming of Adult Ovaries to Testes by FOXL2 Ablation,” Cell 139, no. 6 (2009): 1130–1142, 10.1016/j.cell.2009.11.021. 20005806

[11] A. Georges , B. A. Benayoun , M. Marongiu , et al., “SUMOylation of the Forkhead Transcription Factor FOXL2 Promotes Its Stabilization/Activation Through Transient Recruitment to PML Bodies,” PLoS One 6, no. 10 (2011): e25463, 10.1371/journal.pone.0025463. 22022399 PMC3192040

[12] A. K. Brown and A. E. Webb , “Regulation of FOXO Factors in Mammalian Cells,” Current Topics in Developmental Biology 127 (2018): 165–192, 10.1016/bs.ctdb.2017.10.006. 29433737 PMC6383790

[13] A. Brunet , A. Bonni , M. J. Zigmond , et al., “Akt Promotes Cell Survival by Phosphorylating and Inhibiting a Forkhead Transcription Factor,” Cell 96, no. 6 (1999): 857–868, 10.1016/s0092-8674(00)80595-4. 10102273

[14] G. J. Kops , N. D. de Ruiter , A. M. De Vries‐Smits , D. R. Powell , J. L. Bos , and B. M. Burgering , “Direct Control of the Forkhead Transcription Factor AFX by Protein Kinase B,” Nature 398, no. 6728 (1999): 630–634, 10.1038/19328. 10217147

[15] S. Asada , H. Daitoku , H. Matsuzaki , et al., “Mitogen‐Activated Protein Kinases, Erk and p38, Phosphorylate and Regulate Foxo1,” Cellular Signalling 19, no. 3 (2007): 519–527, 10.1016/j.cellsig.2006.08.015. 17113751

[16] A. Brunet , J. Park , H. Tran , L. S. Hu , B. A. Hemmings , and M. E. Greenberg , “Protein Kinase SGK Mediates Survival Signals by Phosphorylating the Forkhead Transcription Factor FKHRL1 (FOXO3a),” Molecular and Cellular Biology 21, no. 3 (2001): 952–965, 10.1128/MCB.21.3.952-965.2001. 11154281 PMC86685

[17] M. A. G. Essers , S. Weijzen , A. M. M. de Vries‐Smits , et al., “FOXO Transcription Factor Activation by Oxidative Stress Mediated by the Small GTPase Ral and JNK,” EMBO Journal 23, no. 24 (2004): 4802–4812, 10.1038/sj.emboj.7600476. 15538382 PMC535088

[18] E. L. Greer , D. Dowlatshahi , M. R. Banko , et al., “An AMPK‐FOXO Pathway Mediates Longevity Induced by a Novel Method of Dietary Restriction in *C. elegans* ,” Current Biology 17, no. 19 (2007): 1646–1656, 10.1016/j.cub.2007.08.047. 17900900 PMC2185793

[19] M. K. Lehtinen , Z. Yuan , P. R. Boag , et al., “A Conserved MST‐FOXO Signaling Pathway Mediates Oxidative‐Stress Responses and Extends Life Span,” Cell 125, no. 5 (2006): 987–1001, 10.1016/j.cell.2006.03.046. 16751106

[20] M. Boccitto and R. G. Kalb , “Regulation of Foxo‐Dependent Transcription by Post‐Translational Modifications,” Current Drug Targets 12, no. 9 (2011): 1303–1310, 10.2174/138945011796150316. 21443461 PMC3794852

[21] Y. Zhao , Y. Wang , and W. G. Zhu , “Applications of Post‐Translational Modifications of FoxO Family Proteins in Biological Functions,” Journal of Molecular Cell Biology 3, no. 5 (2011): 276–282, 10.1093/jmcb/mjr013. 21669942

[22] M. C. T. Hu , D. F. Lee , W. Xia , et al., “IkappaB Kinase Promotes Tumorigenesis Through Inhibition of Forkhead FOXO3a,” Cell 117, no. 2 (2004): 225–237, 10.1016/s0092-8674(04)00302-2. 15084260

[23] M. D. Pisarska , F. T. Kuo , I. K. Bentsi‐Barnes , S. Khan , and G. M. Barlow , “LATS1 Phosphorylates Forkhead L2 and Regulates Its Transcriptional Activity,” American Journal of Physiology, Endocrinology and Metabolism 299, no. 1 (2010): E101–E109, 10.1152/ajpendo.00534.2009. 20407010 PMC2904049

[24] J. H. Kim , Y. H. Kim , H. M. Kim , et al., “FOXL2 Posttranslational Modifications Mediated by GSK3β Determine the Growth of Granulosa Cell Tumours,” Nature Communications 5 (2014): 2936, 10.1038/ncomms3936. 24390485

[25] Y. Yamashita , M. Okamoto , M. Ikeda , et al., “Protein Kinase C (PKC) Increases TACE/ADAM17 Enzyme Activity in Porcine Ovarian Somatic Cells, Which Is Essential for Granulosa Cell Luteinization and Oocyte Maturation,” Endocrinology 155, no. 3 (2014): 1080–1090, 10.1210/en.2013-1655. 24424050

[26] F. Tepekoy , I. Ustunel , and G. Akkoyunlu , “Protein Kinase C Isoforms α, δ and ε Are Differentially Expressed in Mouse Ovaries at Different Stages of Postnatal Development,” Journal of Ovarian Research 7 (2014): 117, 10.1186/s13048-014-0117-z. 25491605 PMC4271327

[27] D. González‐Aretia , C. G. Hernández‐Coronado , A. Guzmán , Z. B. Medina‐Moctezuma , C. G. Gutiérrez , and A. M. Rosales‐Torres , “Sphingosine‐1‐Phosphate Mediates FSH‐Induced Cell Viability but Not Steroidogenesis in Bovine Granulosa Cells,” Theriogenology 213 (2024): 90–96, 10.1016/j.theriogenology.2023.10.003. 37820497

[28] P. G. Tremblay and M. A. Sirard , “Gene Analysis of Major Signaling Pathways Regulated by Gonadotropins in Human Ovarian Granulosa Tumor Cells (KGN)†,” Biology of Reproduction 103, no. 3 (2020): 583–598, 10.1093/biolre/ioaa079. 32427331

[29] L. Herman , A. Amo , B. Legois , C. Di Carlo , R. A. Veitia , and A. L. Todeschini , “A Cellular Model Provides Insights Into the Pathogenicity of the Oncogenic FOXL2 Somatic Variant p.Cys134Trp,” British Journal of Cancer 130, no. 9 (2024): 1453–1462, 10.1038/s41416-024-02613-x. 38429437 PMC11059147

[30] L. Moumné , A. Dipietromaria , F. Batista , et al., “Differential Aggregation and Functional Impairment Induced by Polyalanine Expansions in FOXL2, a Transcription Factor Involved in Cranio‐Facial and Ovarian Development,” Human Molecular Genetics 17, no. 7 (2008): 1010–1019, 10.1093/hmg/ddm373. 18158309

[31] R. Higuchi , B. Krummel , and R. K. Saiki , “A General Method of In Vitro Preparation and Specific Mutagenesis of DNA Fragments: Study of Protein and DNA Interactions,” Nucleic Acids Research 16, no. 15 (1988): 7351–7367, 10.1093/nar/16.15.7351. 3045756 PMC338413

[32] R. Zufferey , D. Nagy , R. J. Mandel , L. Naldini , and D. Trono , “Multiply Attenuated Lentiviral Vector Achieves Efficient Gene Delivery In Vivo,” Nature Biotechnology 15, no. 9 (1997): 871–875, 10.1038/nbt0997-871. 9306402

[33] M. Kwon and B. L. Firestein , “DNA Transfection: Calcium Phosphate Method,” Methods in Molecular Biology 1018 (2013): 107–110, 10.1007/978-1-62703-444-9_10. 23681621

[34] G. Koulouras , A. Panagopoulos , M. A. Rapsomaniki , N. N. Giakoumakis , S. Taraviras , and Z. Lygerou , “EasyFRAP‐Web: A Web‐Based Tool for the Analysis of Fluorescence Recovery After Photobleaching Data,” Nucleic Acids Research 46, no. W1 (2018): W467–W472, 10.1093/nar/gky508. 29901776 PMC6030846

[35] S. Perrin , C. Firmo , S. Lemoine , S. Le Crom , and L. Jourdren , “Aozan: An Automated Post‐Sequencing Data‐Processing Pipeline,” Bioinformatics 33, no. 14 (2017): 2212–2213, 10.1093/bioinformatics/btx154. 28369225

[36] C. Zutterling , A. L. Todeschini , D. Fourmy , et al., “The Forkhead DNA‐Binding Domain Binds Specific G2‐Rich RNA Sequences,” Nucleic Acids Research 51, no. 22 (2023): 12367–12380, 10.1093/nar/gkad994. 37933840 PMC10711433

[37] A. L. Todeschini , A. Dipietromaria , D. L'hôte , et al., “Mutational Probing of the Forkhead Domain of the Transcription Factor FOXL2 Provides Insights Into the Pathogenicity of Naturally Occurring Mutations,” Human Molecular Genetics 20, no. 17 (2011): 3376–3385, 10.1093/hmg/ddr244. 21632871

[38] D. L'Hôte , A. Georges , A. L. Todeschini , et al., “Discovery of Novel Protein Partners of the Transcription Factor FOXL2 Provides Insights Into Its Physiopathological Roles,” Human Molecular Genetics 21, no. 14 (2012): 3264–3274, 10.1093/hmg/dds170. 22544055

[39] B. A. Benayoun , F. Batista , J. Auer , et al., “Positive and Negative Feedback Regulates the Transcription Factor FOXL2 in Response to Cell Stress: Evidence for a Regulatory Imbalance Induced by Disease‐Causing Mutations,” Human Molecular Genetics 18, no. 4 (2009): 632–644, 10.1093/hmg/ddn389. 19010791

[40] B. S. Ellsworth , A. T. Burns , K. W. Escudero , D. L. Duval , S. E. Nelson , and C. M. Clay , “The Gonadotropin Releasing Hormone (GnRH) Receptor Activating Sequence (GRAS) is a Composite Regulatory Element That Interacts With Multiple Classes of Transcription Factors Including Smads, AP‐1 and a Forkhead DNA Binding Protein,” Molecular and Cellular Endocrinology 206, no. 1–2 (2003): 93–111, 10.1016/s0303-7207(03)00235-1. 12943993

[41] D. Beysen , L. Moumné , R. Veitia , et al., “Missense Mutations in the Forkhead Domain of FOXL2 Lead to Subcellular Mislocalization, Protein Aggregation and Impaired Transactivation,” Human Molecular Genetics 17, no. 13 (2008): 2030–2038, 10.1093/hmg/ddn100. 18372316

[42] M. M. Brent , R. Anand , and R. Marmorstein , “Structural Basis for DNA Recognition by FoxO1 and Its Regulation by Posttranslational Modification,” Structure 16, no. 9 (2008): 1407–1416, 10.1016/j.str.2008.06.013. 18786403 PMC2597217

[43] A. Georges , D. L'Hôte , A. L. Todeschini , et al., “The Transcription Factor FOXL2 Mobilizes Estrogen Signaling to Maintain the Identity of Ovarian Granulosa Cells,” eLife 3 (2014): e04207, 10.7554/eLife.04207. 25369636 PMC4356143

[44] F. T. Kuo , I. K. Bentsi‐Barnes , G. M. Barlow , and M. D. Pisarska , “Mutant Forkhead L2 (FOXL2) Proteins Associated With Premature Ovarian Failure (POF) Dimerize With Wild‐Type FOXL2, Leading to Altered Regulation of Genes Associated With Granulosa Cell Differentiation,” Endocrinology 152, no. 10 (2011): 3917–3929, 10.1210/en.2010-0989. 21862621 PMC3176639

[45] L. Herman , B. Legois , A. L. Todeschini , and R. A. Veitia , “Genomic Exploration of the Targets of FOXL2 and ESR2 Unveils Their Implication in Cell Migration, Invasion, and Adhesion,” FASEB Journal 35, no. 4 (2021): e21355, 10.1096/fj.202002444R. 33749886

[46] Z. Xie , A. Bailey , M. V. Kuleshov , et al., “Gene Set Knowledge Discovery With Enrichr,” Current Protocols 1, no. 3 (2021): e90, 10.1002/cpz1.90. 33780170 PMC8152575

[47] E. Y. Chen , C. M. Tan , Y. Kou , et al., “Enrichr: Interactive and Collaborative HTML5 Gene List Enrichment Analysis Tool,” BMC Bioinformatics 14 (2013): 128, 10.1186/1471-2105-14-128. 23586463 PMC3637064

[48] M. V. Kuleshov , M. R. Jones , A. D. Rouillard , et al., “Enrichr: A Comprehensive Gene Set Enrichment Analysis Web Server 2016 Update,” Nucleic Acids Research 44, no. W1 (2016): W90–W97, 10.1093/nar/gkw377. 27141961 PMC4987924

[49] M. Penrad‐Mobayed , C. Perrin , L. Herman , et al., “Conventional and Unconventional Interactions of the Transcription Factor FOXL2 Uncovered by a Proteome‐Wide Analysis,” FASEB Journal 34, no. 1 (2020): 571–587, 10.1096/fj.201901573R. 31914586

[50] L. Stenström , D. Mahdessian , C. Gnann , et al., “Mapping the Nucleolar Proteome Reveals a Spatiotemporal Organization Related to Intrinsic Protein Disorder,” Molecular Systems Biology 16, no. 8 (2020): e9469, 10.15252/msb.20209469. 32744794 PMC7397901

[51] A. Salvetti , Y. Couté , A. Epstein , et al., “Nuclear Functions of Nucleolin Through Global Proteomics and Interactomic Approaches,” Journal of Proteome Research 15, no. 5 (2016): 1659–1669, 10.1021/acs.jproteome.6b00126. 27049334

[52] R. Cong , S. Das , J. Douet , et al., “macroH2A1 Histone Variant Represses rDNA Transcription,” Nucleic Acids Research 42, no. 1 (2014): 181–192, 10.1093/nar/gkt863. 24071584 PMC3874179

[53] N. Hamdane , V. Y. Stefanovsky , M. G. Tremblay , et al., “Conditional Inactivation of Upstream Binding Factor Reveals Its Epigenetic Functions and the Existence of a Somatic Nucleolar Precursor Body,” PLoS Genetics 10, no. 8 (2014): e1004505, 10.1371/journal.pgen.1004505. 25121932 PMC4133168

[54] N. Duployez , L. Vasseur , R. Kim , et al., “UBTF Tandem Duplications Define a Distinct Subtype of Adult De Novo Acute Myeloid Leukemia,” Leukemia 37, no. 6 (2023): 1245–1253, 10.1038/s41375-023-01906-z. 37085611 PMC10244165

[55] Z. Wang , L. Bai , Y. J. Hsieh , and R. G. Roeder , “Nuclear Factor 1 (NF1) Affects Accurate Termination and Multiple‐Round Transcription by Human RNA Polymerase III,” EMBO Journal 19, no. 24 (2000): 6823–6832, 10.1093/emboj/19.24.6823. 11118217 PMC305894

[56] X. Li , W. Wang , J. Wang , et al., “Proteomic Analyses Reveal Distinct Chromatin‐Associated and Soluble Transcription Factor Complexes,” Molecular Systems Biology 11, no. 1 (2015): 775, 10.15252/msb.20145504. 25609649 PMC4332150

[57] M. Ciesla , E. Skowronek , and M. Boguta , “Function of TFIIIC, RNA Polymerase III Initiation Factor, in Activation and Repression of tRNA Gene Transcription,” Nucleic Acids Research 46, no. 18 (2018): 9444–9455, 10.1093/nar/gky656. 30053100 PMC6182151

[58] S. Brodsky , T. Jana , K. Mittelman , et al., “Intrinsically Disordered Regions Direct Transcription Factor In Vivo Binding Specificity,” Molecular Cell 79, no. 3 (2020): 459–471.e4, 10.1016/j.molcel.2020.05.032. 32553192

[59] Y. Zhang , X. Liang , S. Luo , et al., “Visualizing the Nucleoplasmic Maturation of Human Pre‐60S Ribosomal Particles,” Cell Research 33, no. 11 (2023): 867–878, 10.1038/s41422-023-00853-9. 37491604 PMC10624882

[60] S. K. Kim , H. Lee , K. Han , et al., “SET7/9 Methylation of the Pluripotency Factor LIN28A Is a Nucleolar Localization Mechanism That Blocks Let‐7 Biogenesis in Human ESCs,” Cell Stem Cell 15, no. 6 (2014): 735–749, 10.1016/j.stem.2014.10.016. 25479749 PMC4258232

