# Supplementary material for: Modification of the Transcription Factor FOXL2 at Serines 101 and 107 Disables DNA Binding, Leads to Nucleolar Relocalization, and Rewires Granulosa‐Cell Programs
Source: FASEB J. 2026 Aug 1;40(15):e72177. doi: 10.1096/fj.202602528R (PMC13428323; doi:10.1096/fj.202602528R)
Supplement: Supplementary file 1 — Figure S1: Intact mass analysis of FOXL2 reveals phosphorylated proteoforms. Deconvoluted intact mass spectrum of recombinant FOXL2 analyzed by TIMS‐TOF mass spectrometry. Peak intensities are reported as relative signal abundance after deconvolution of the charge‐state distribution. The experimental mass of the unmodified protein was measured at 38 768.6 Da, in agreement with the theoretical mass of 38 765.6 Da (ΔM = 2.97 Da). Additional proteoforms were detected with mass increases consistent with the presence of one (+80 Da) and two (+160 Da) phosphorylation events, yielding experimental masses of 38 848.7 Da and 38 930.3 Da, respectively. The observed mass shifts correspond to the addition of phosphate groups (+79.97 Da each), confirming the occurrence of multiple phosphorylation states of FOXL2 by PKC in vitro. Figure S2: Post‐translational modification mapping of FOXL2 obtained by multi‐enzymatic digestion and mass spectrometry for PKC. FOXL2 was digested using trypsin and chymotrypsin. The resulting peptides were analyzed by LC–MS/MS. Blue bars represent the identified peptides mapped onto the FOXL2 amino acid sequence. Detected post‐translational modifications are indicated above the sequence. The Serine phosphorylated by PKC are highlighted with red rectangles. Figure S3: Luciferase assays in HeLa cells transfected with (A) GRAS‐luc, (B) p16‐luc and (C) pAT29C‐luc, respectively, with or without overexpression of WT FOXL2 or the mutated forms. For each PKC‐phosphorylation site identified within the FOXL2 forkhead domain, serine residues were replaced by alanine (A) to mimic the unphosphorylated state and by aspartate (D) to mimic the phosphorylated state. Each value is representative of six biological replicates. Errors bars represent SD. Letters A, B, C, D, E and F refer to statistical categories in a Tukey test. Figure S4: All mutants are mobile. HeLa cells were transiently transfected with constructs expressing WT FOXL2 or FOXL2 variants fused to GFP. Flu [file FSB2-40-e72177-s002.pptx]

## Slide 1
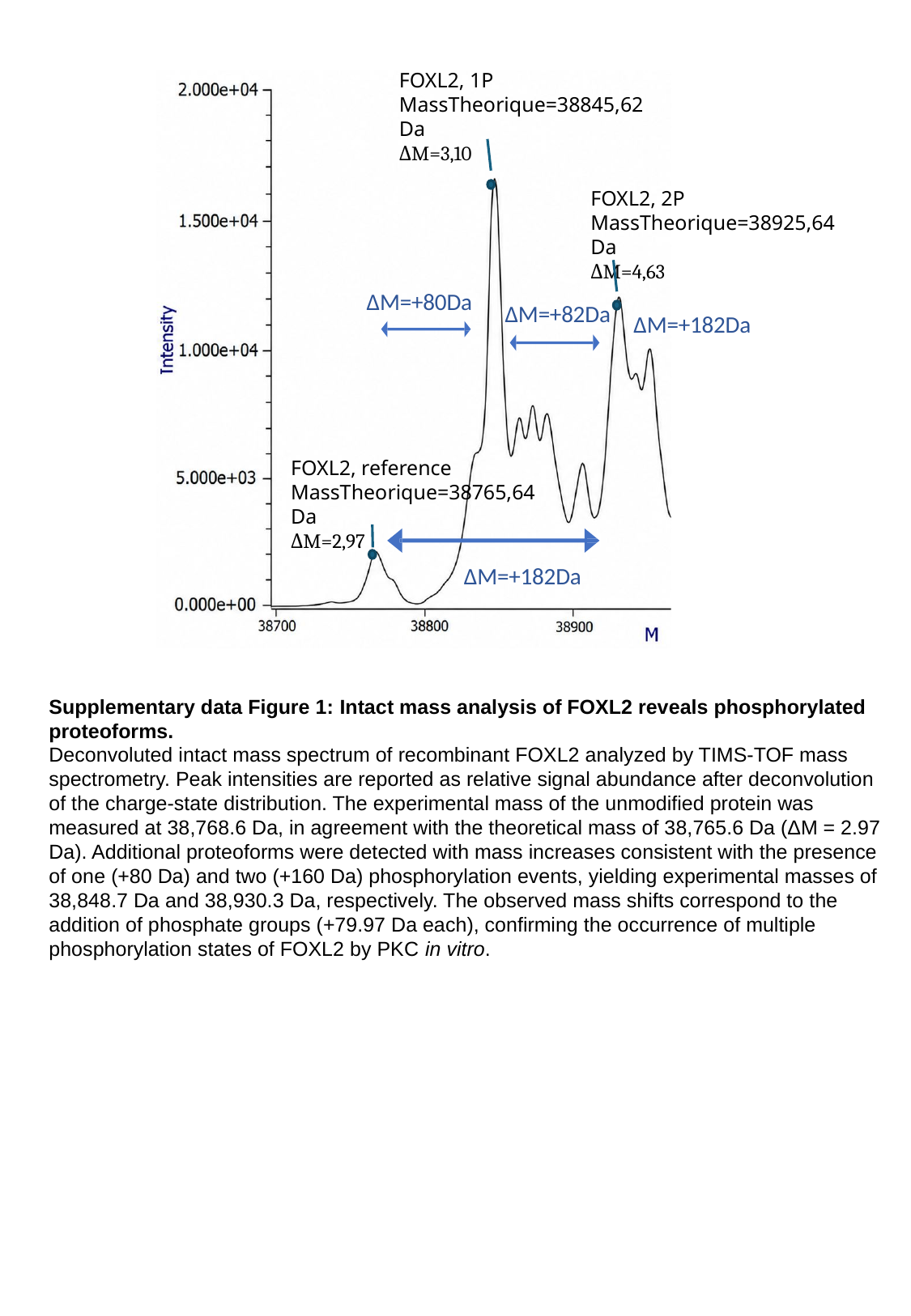

FOXL2, 1P
MassTheorique=38845,62Da
ΔM=3,10
FOXL2, 2P
MassTheorique=38925,64Da
ΔM=4,63
∆M=+80Da
∆M=+82Da
∆M=+182Da
FOXL2, reference
MassTheorique=38765,64Da
ΔM=2,97
∆M=+82Da
∆M=+80Da
∆M=+182Da
Supplementary data Figure 1: Intact mass analysis of FOXL2 reveals phosphorylated proteoforms.
Deconvoluted intact mass spectrum of recombinant FOXL2 analyzed by TIMS-TOF mass spectrometry. Peak intensities are reported as relative signal abundance after deconvolution of the charge-state distribution. The experimental mass of the unmodified protein was measured at 38,768.6 Da, in agreement with the theoretical mass of 38,765.6 Da (ΔM = 2.97 Da). Additional proteoforms were detected with mass increases consistent with the presence of one (+80 Da) and two (+160 Da) phosphorylation events, yielding experimental masses of 38,848.7 Da and 38,930.3 Da, respectively. The observed mass shifts correspond to the addition of phosphate groups (+79.97 Da each), confirming the occurrence of multiple phosphorylation states of FOXL2 by PKC in vitro.

## Slide 2
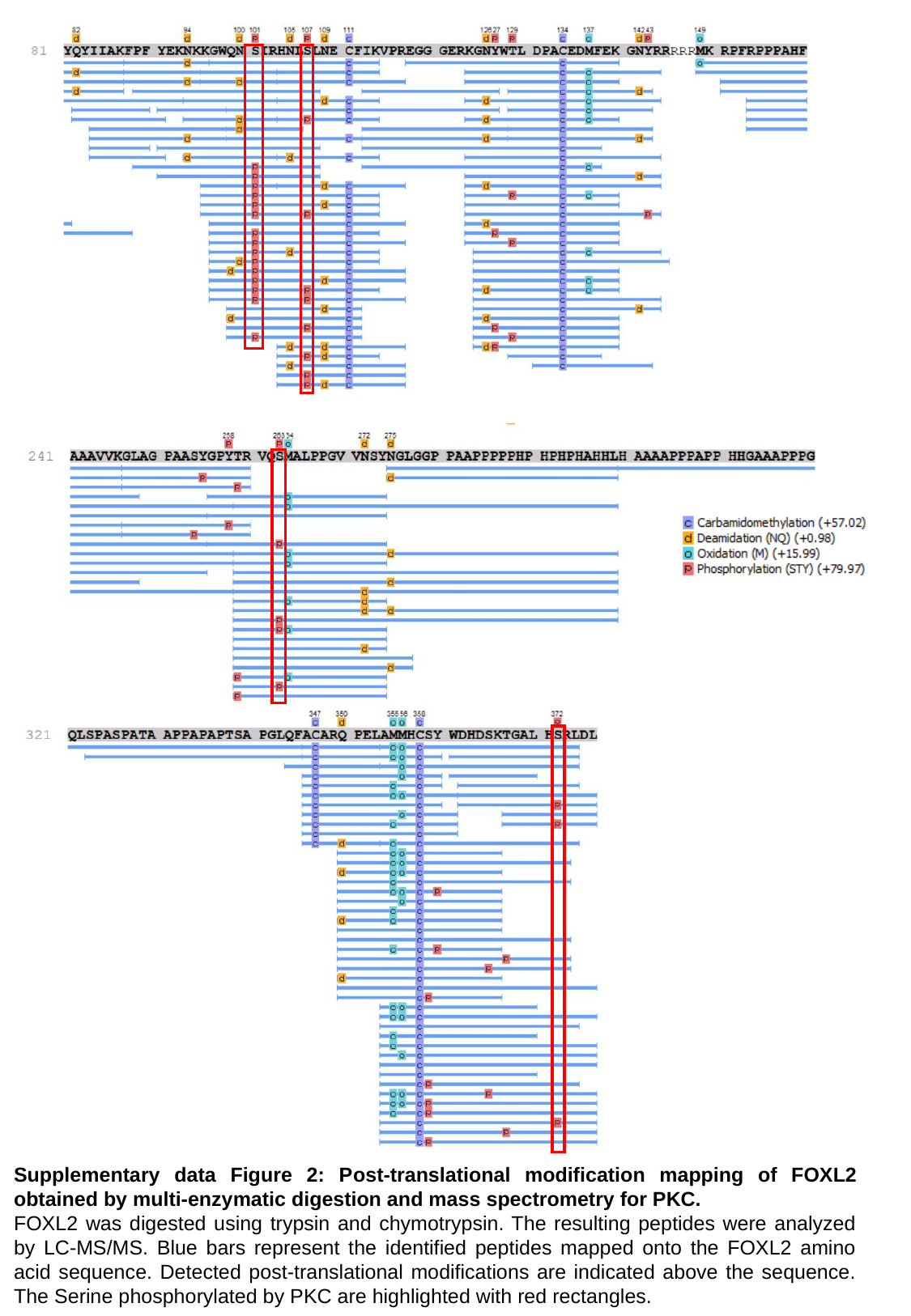

Supplementary data Figure 2: Post-translational modification mapping of FOXL2 obtained by multi-enzymatic digestion and mass spectrometry for PKC.
FOXL2 was digested using trypsin and chymotrypsin. The resulting peptides were analyzed by LC-MS/MS. Blue bars represent the identified peptides mapped onto the FOXL2 amino acid sequence. Detected post-translational modifications are indicated above the sequence. The Serine phosphorylated by PKC are highlighted with red rectangles.

## Slide 3
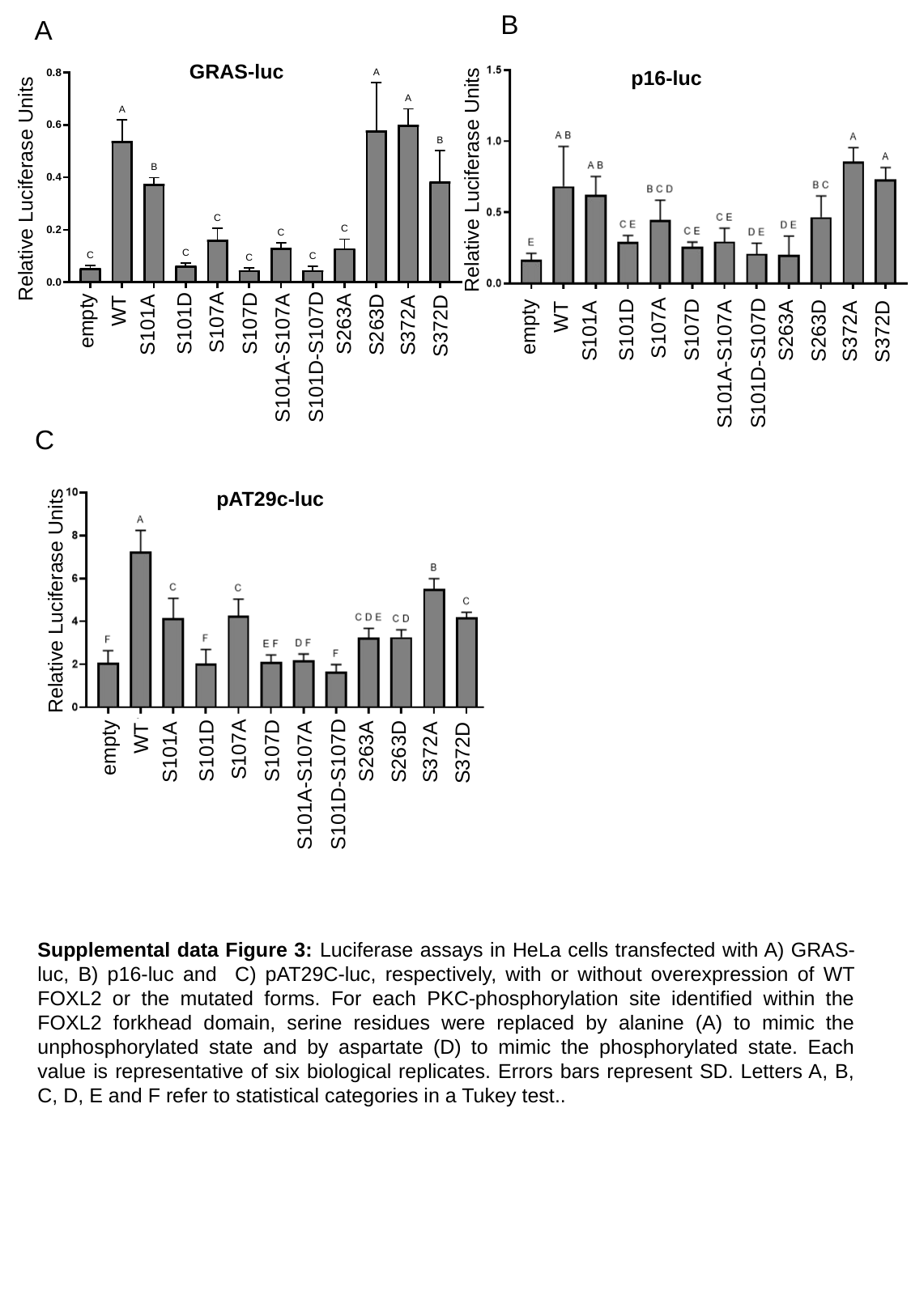

B
A
GRAS-luc
Relative Luciferase Units
WT
empty
S107A
S101D
S107D
S101A
S263A
S263D
S372A
S372D
S101A-S107A
S101D-S107D
p16-luc
Relative Luciferase Units
WT
empty
S107A
S101D
S107D
S101A
S263A
S263D
S372A
S372D
S101A-S107A
S101D-S107D
C
pAT29c-luc
Relative Luciferase Units
WT
empty
S107A
S101D
S107D
S101A
S263A
S263D
S372A
S372D
S101A-S107A
S101D-S107D
Supplemental data Figure 3: Luciferase assays in HeLa cells transfected with A) GRAS-luc, B) p16-luc and C) pAT29C-luc, respectively, with or without overexpression of WT FOXL2 or the mutated forms. For each PKC-phosphorylation site identified within the FOXL2 forkhead domain, serine residues were replaced by alanine (A) to mimic the unphosphorylated state and by aspartate (D) to mimic the phosphorylated state. Each value is representative of six biological replicates. Errors bars represent SD. Letters A, B, C, D, E and F refer to statistical categories in a Tukey test..

## Slide 4
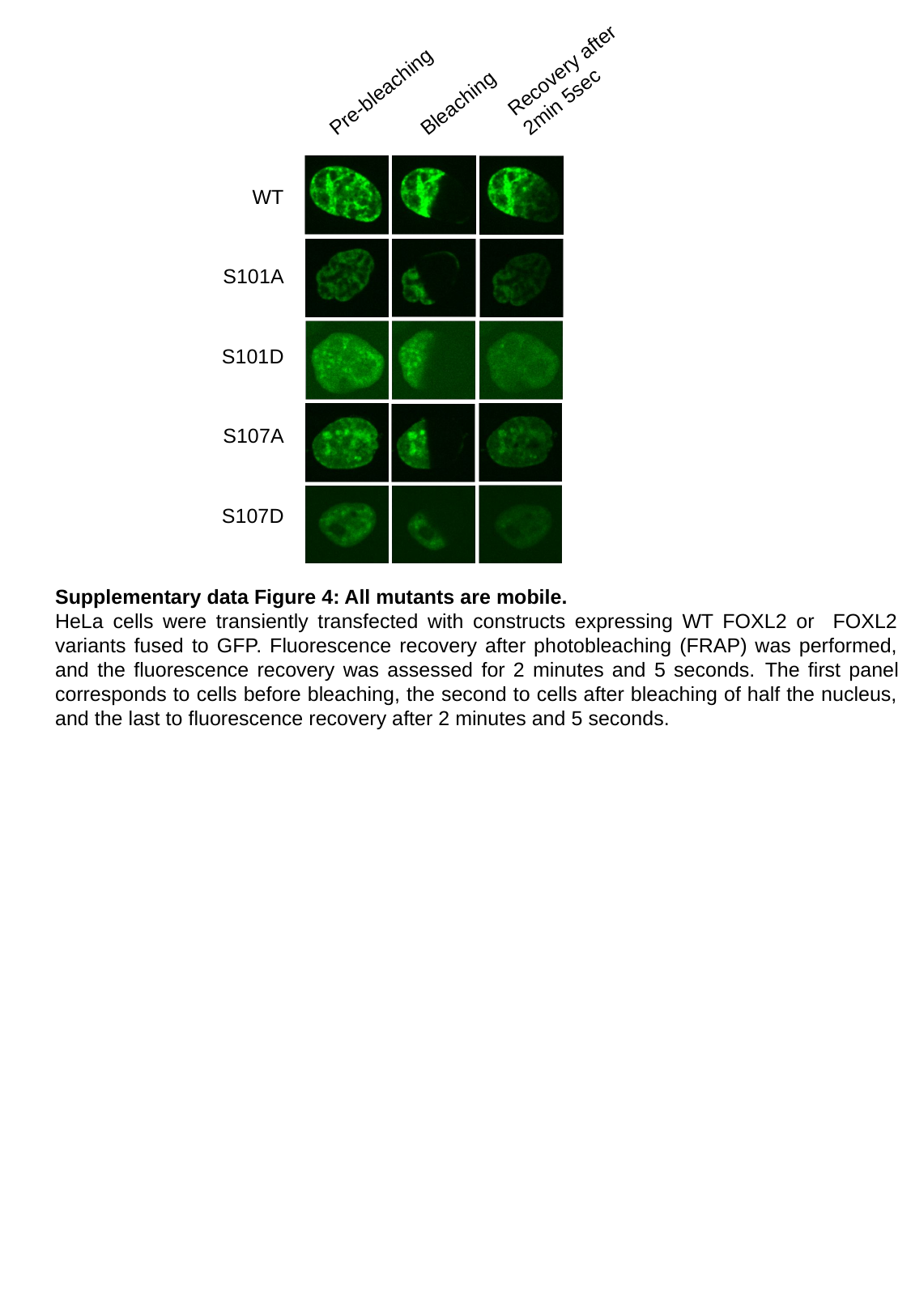

Recovery after
2min 5sec
Pre-bleaching
Bleaching
WT
S101A
S101D
S107A
S107D
Supplementary data Figure 4: All mutants are mobile.
HeLa cells were transiently transfected with constructs expressing WT FOXL2 or FOXL2 variants fused to GFP. Fluorescence recovery after photobleaching (FRAP) was performed, and the fluorescence recovery was assessed for 2 minutes and 5 seconds. The first panel corresponds to cells before bleaching, the second to cells after bleaching of half the nucleus, and the last to fluorescence recovery after 2 minutes and 5 seconds.

## Slide 5
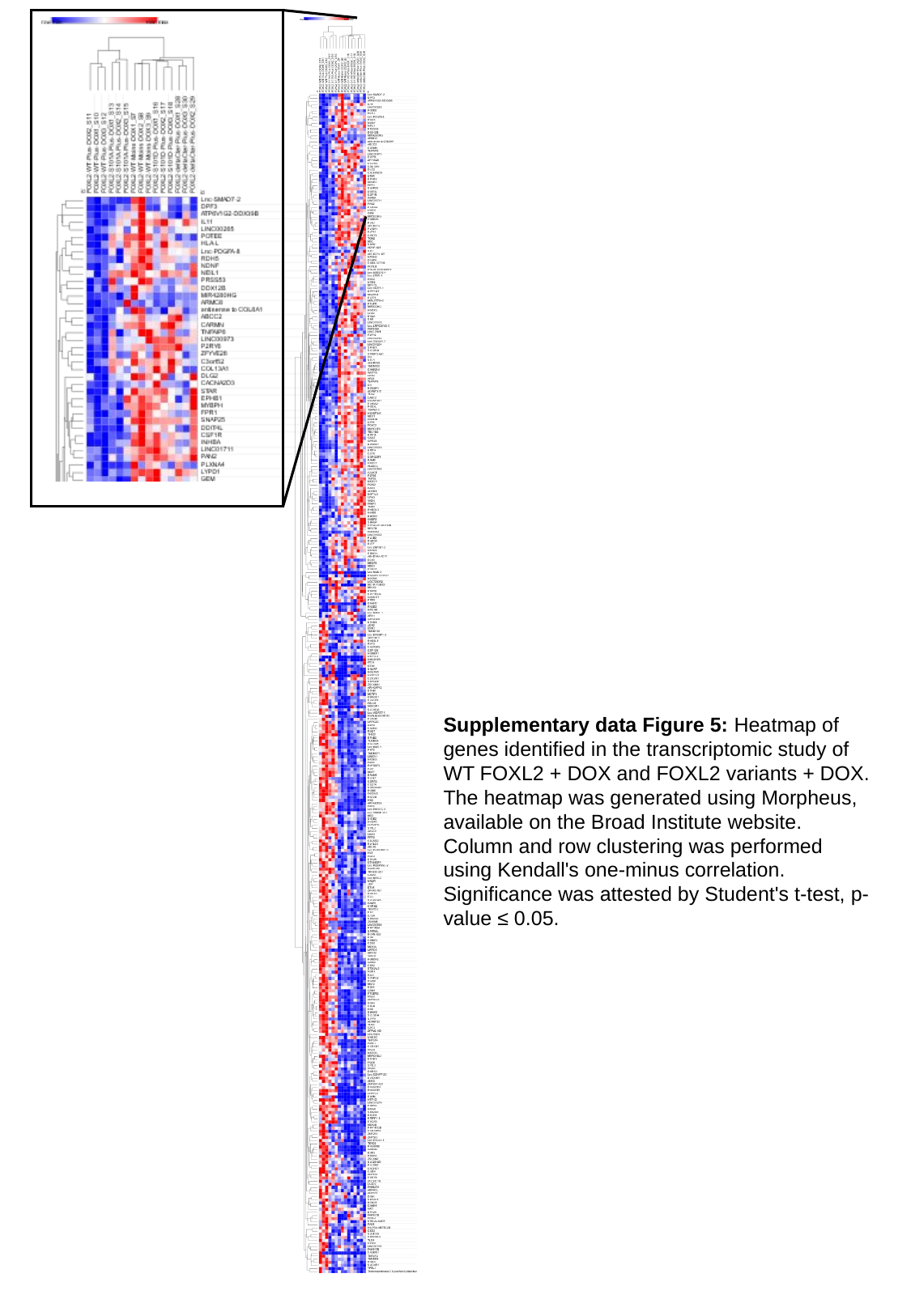

Supplementary data Figure 5: Heatmap of genes identified in the transcriptomic study of WT FOXL2 + DOX and FOXL2 variants + DOX. The heatmap was generated using Morpheus, available on the Broad Institute website. Column and row clustering was performed using Kendall's one-minus correlation. Significance was attested by Student's t-test, p-value ≤ 0.05.

## Slide 6
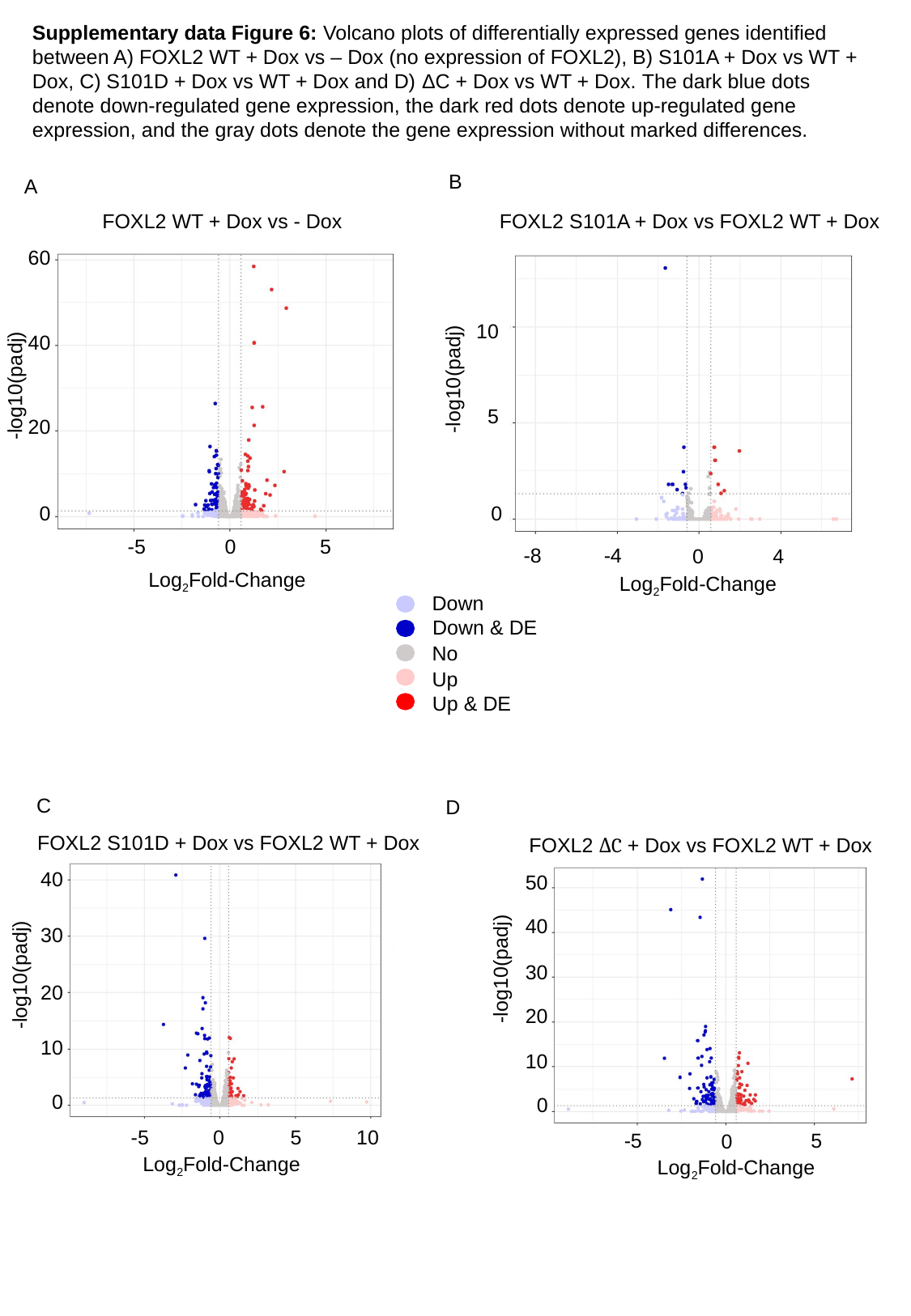

Supplementary data Figure 6: Volcano plots of differentially expressed genes identified between A) FOXL2 WT + Dox vs – Dox (no expression of FOXL2), B) S101A + Dox vs WT + Dox, C) S101D + Dox vs WT + Dox and D) ΔC + Dox vs WT + Dox. The dark blue dots denote down-regulated gene expression, the dark red dots denote up-regulated gene expression, and the gray dots denote the gene expression without marked differences.
B
A
FOXL2 S101A + Dox vs FOXL2 WT + Dox
FOXL2 WT + Dox vs - Dox
60
10
40
-log10(padj)
-log10(padj)
5
20
0
0
-5
0
5
-8
-4
0
4
Log2Fold-Change
Log2Fold-Change
Down
Down & DE
No
Up
Up & DE
C
D
FOXL2 S101D + Dox vs FOXL2 WT + Dox
FOXL2 ΔC + Dox vs FOXL2 WT + Dox
40
50
40
30
-log10(padj)
30
-log10(padj)
20
20
10
10
0
0
-5
0
5
10
-5
5
0
Log2Fold-Change
Log2Fold-Change

## Slide 7
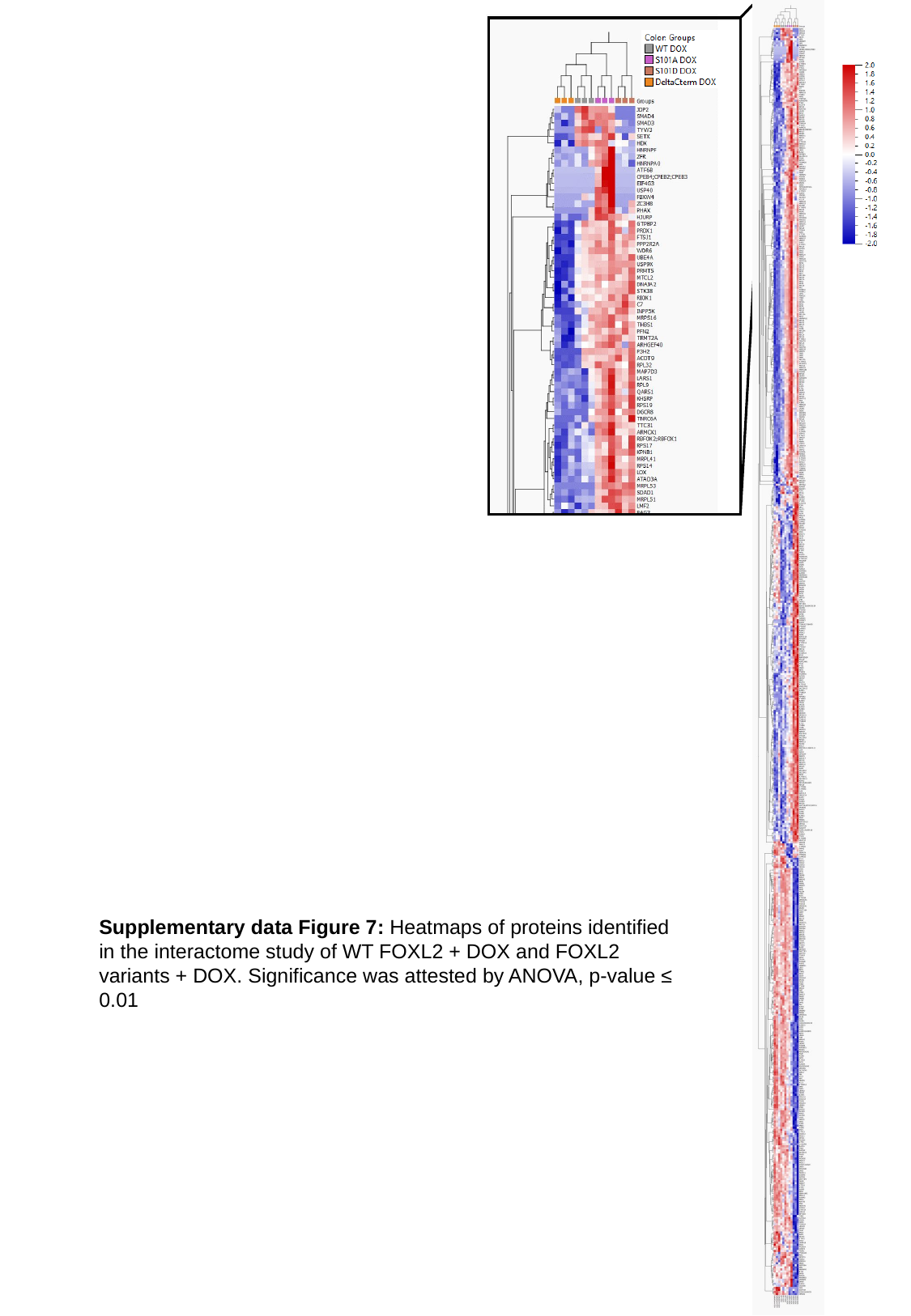

Supplementary data Figure 7: Heatmaps of proteins identified in the interactome study of WT FOXL2 + DOX and FOXL2 variants + DOX. Significance was attested by ANOVA, p-value ≤ 0.01
